# Lipidomic and Fatty Acid Biomarkers in Whole Blood Can Predict the Dietary Intake of Eicosapentaenoic and Docosahexaenoic Acids in a Danish Population

**DOI:** 10.1016/j.tjnut.2024.04.038

**Published:** 2024-05-04

**Authors:** Juan J Aristizabal-Henao, Anja P Biltoft-Jensen, Tue Christensen, Ken D Stark

**Affiliations:** 1 Department of Kinesiology and Health Sciences, University of Waterloo, Waterloo, ON, Canada; 2 Platforms and Translational Sciences, BPGbio Inc., Framingham, MA, United States; 3 National Food Institute, Technical University of Denmark, Kongens Lyngby, Denmark

**Keywords:** dietary assessment, omega-3 polyunsaturated fatty acids, lipidomics, blood biomarkers, dietary intake, eicosapentaenoic acid, docosahexaenoic acid, gas chromatography, tandem mass spectrometry

## Abstract

**Background:**

The intake of eicosapentaenoic acid (EPA) and docosahexaenoic acid (DHA) have been associated with health benefits. Blood levels of these fatty acids, measured by gas chromatography (GC), are associated with their dietary intake, but the relationships with lipidomic measurements are not well defined.

**Objectives:**

This study aimed to determine the lipidomic biomarkers in whole blood that predict intakes of EPA + DHA and examine the relationship between lipidomic and GC-based n–3 polyunsaturated fatty acid (n–3 PUFA) biomarkers.

**Methods:**

Lipidomic and fatty acid analyses were completed on 120 whole blood samples collected from Danish participants. Dietary intakes were completed using a web-based 7-d food diary. Stepwise multiple linear regression was used to identify the fatty acid and lipidomic variables that predict intakes of EPA + DHA and to determine lipidomic species that predict commonly used fatty acid biomarkers.

**Results:**

Stepwise regression selected lipidomic variables with an *R*^2^ = 0.52 for predicting EPA + DHA intake compared to *R*^2^ = 0.40 for the selected fatty acid GC-based variables. More predictive models were generated when the lipidomic variables were selected for females only (*R*^2^ = 0.62, *n* = 68) and males only (*R*^2^ = 0.72, *n* = 52). Phosphatidylethanolamine plasmalogen species containing EPA or DHA tended to be the most predictive lipidomic variables. Stepwise regression also indicated that selected lipidomic variables can predict commonly used fatty acid GC-based n–3 PUFA biomarkers as the *R*^2^ values ranged from 0.84 to 0.91.

**Conclusions:**

Both fatty acid and lipidomic data can be used to predict EPA + DHA intakes, and fatty acid GC-based biomarkers can be emulated by lipidomic species. Lipidomic-based biomarkers appear to be influenced by sex differences, probably in n–3 PUFA and lipoprotein metabolism. These results improve our ability to understand the relationship between novel lipidomic data and GC fatty acid data and will increase our ability to apply lipidomic methods to fatty acid and lipid nutritional research.

## Graphical abstract

**Figure:**
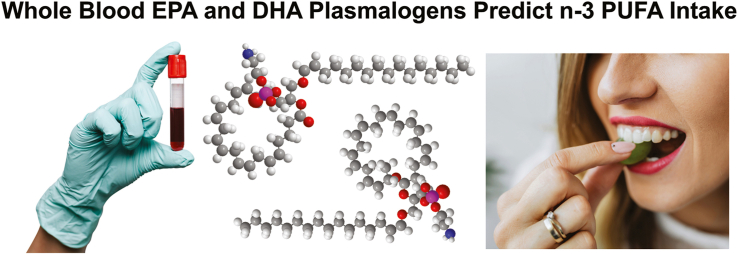

## Introduction

Dietary intakes of the n–3 polyunsaturated fatty acids (n–3 PUFAs) EPA (20:5n–3) and DHA (22:6n–3) have been associated with a decreased risk of cardiovascular disease [1], cognitive decline [2], and preterm birth [3]. Assessing dietary intake is challenging in general and subject to random and systematic measurement error [4]. Specifically, in many Westernized populations, foods containing high amounts of EPA and/or DHA are consumed sporadically with considerable day to day variation that can introduce significant error in estimating usual intake from short-term assessments such as food records and dietary recalls [5,6]. EPA- and DHA-containing foods are also at risk of overreporting or increased intake during the self-report period due to the social desirability and/or social approval to report the intake of healthy foods [7]. Due to the challenges in measuring dietary intakes, measuring biomarkers of EPA and DHA intake has been recommended for research involving fatty acids [8,9].

The relationship between the dietary intake of n–3 PUFA to the fatty acid compositions of tissues and blood levels is rooted in the early work by Mohrhauer and Holman [10] with empirical equations for predicting the long-chain PUFA (LCPUFA) composition of plasma phospholipids from dietary intake information being developed by Lands et al. [11]. Various fatty acid-based biomarkers from different tissues and blood fractions have been proposed and developed [8,[12], [13], [14]], with the percentage of EPA + DHA in erythrocytes or the Omega-3 Index [15] becoming one of the most popular. These biomarkers can be used for estimating dietary intakes of EPA and DHA [7,16] or for stratifying disease risk [15,17]. How to define these n–3 PUFA biomarkers and which ones to use for different applications in various clinical populations continues to be debated [18,19].

Measures of EPA and DHA and other fatty acids in blood have traditionally been completed using gas chromatography with flame ionization detection (GC-FID)-based analyses. Information about lipid classes and individual lipid species are lost during sample preparation for GC-FID because fatty acyl chains are removed from complex lipids and derivatized to form fatty acid methyl esters [20]. The resulting fatty acid composition data can be presented quantitatively (as concentrations) and/or qualitatively (as the percentage of total fatty acids) [8]. Regardless, the chemical processing and isolation of the fatty acids from their original lipids limits the ability to characterize the complex *in vivo* lipid structures, and specific structural information of lipids that could be of physiological relevance is lost. Lipidomic analyses using ultra high-performance liquid chromatography coupled to tandem mass spectrometry (UHPLC-MS/MS) can identify complex lipids as they exist in their natural states but with much more analytical burden and cost than GC-FID [8]. Lipidomic characterization of blood samples, however, can enhance our understanding of the effect of diet on lipid metabolism and has the potential to identify novel and more informative biomarkers of dietary intake and n–3 PUFA status in the body.

In the present study, lipidomics and traditional fatty acid analyses were completed on 120 whole blood samples collected as part of an evaluation of dietary assessment methods for the Danish National Survey of Dietary Habits and Physical Activity [21]. The relationships between dietary intake estimates of n–3 PUFA with lipidomic and fatty acid (quantitative and qualitative) measures in human whole blood were examined using a series of stepwise linear regression models. This approach allows for the most predictive blood biomarker variables within an analytical approach to be identified but also allows for comparison of the predictive models across the analytical approach. Given that sex differences [9] and the use of fish oil supplements [22] are known to impact blood fatty acid status, analyses comparing males and females and those taking and not taking fish oil supplements were explored. Stepwise linear regression was also used to identify the lipid acyl species as determined by lipidomic analyses that predict established and commonly used gas chromatography-based fatty acid biomarkers of n–3 PUFA status.

## Methods

### Participants and study design

The present analyses were completed on blood samples collected for a validation of a 2 × 24-h dietary recall method and a 7-d web-based food diary for the Danish National Survey of Diet and Physical Activity conducted at the National Food Institute, Technical University of Denmark that has been described previously [21]. Blood fatty acid measurements are commonly used to validate the ability of food surveys to estimate dietary intakes of fatty acids [6,[23], [24], [25], [26]]. Briefly, the blood samples were collected after the 2 dietary assessment methods had been completed at the final visit of a 4-wk crossover study. Overnight fasting blood samples were collected from venous blood drawn from the forearm into EDTA tubes and gently mixed, and the whole blood was aliquoted and stored at −80°C until shipment on dry ice to the University of Waterloo where the samples were kept at −80 ºC until analyses. A total of 120 volunteers aged 18 to 60 y were recruited from the local area around The Technical University of Denmark. An equal distribution of males and females in the age ranges of 18 to 30, 31 to 45, and 46 to 60 y was established. Inclusion criteria were weight stable Danish speakers that had access to the Internet, with no chronic disease requiring medicine. Pregnant and lactating females and nutrition professionals were also excluded.

All participants entering the study provided written informed consent, and the study was conducted according to the guidelines stated in the Declaration of Helsinki. All procedures involving human participants were approved by the Regional Ethical Committee of Copenhagen and by the Danish Data Protection Agency (no. 17006825). Sample fatty acid and lipidomic analyses were also ethically approved by the University of Waterloo Office of Research Ethics.

### Measures

#### Dietary intake estimates

Dietary intakes for all participants were assessed using a web-based 7-d food diary [23,24]. The food diary guided participants to self-report intakes through 6 daily eating occasions (breakfast, morning snack, lunch, afternoon snack, dinner, and evening snack) for 7 consecutive days. Participants could search the existing database or type in foods, and portion sizes were selected with the assistance of digital images. Participants were prompted to check for frequently forgotten foods, and follow-up emails and phone calls were used when participants failed to report for a day. Fish oil supplementation use was also documented, and n–3 PUFA intakes from these supplements were estimated based on commercial data of fish oil brands in the Danish market. To be included in the analyses, participants had to complete ≥4 d including 1 weekend day. The macronutrients, fatty acid classes, and selected individual fatty acids were calculated using the Danish Food Composition Database Frida (https://frida.fooddata.dk), version 3; Søborg, Denmark; 23-03-2018.

#### Lipid extraction

Lipids were extracted from whole blood by adding 2:1 chloroform/methanol (v/v) that contained either known amounts of deuterium-labeled internal standards for the major lipid classes (Splash Lipidomix, Avanti Polar Lipids) for lipidomic analyses or docosatrienoate methyl ester internal standard (NuChek-Prep) for fatty acid analyses. Samples were then vortexed, and the addition of saline buffer allowed the organic layer to be isolated and collected [25]. Additional chloroform was added to the remaining aqueous material followed by vortexing and centrifugation to allow a second organic layer to be collected that was then combined with the original collection.

#### Fatty acid analyses

Whole blood fatty acids were determined by GC-FID as described previously [26]. Lipid extracts were dried under a gentle stream of N_2_ gas and then derivatized to fatty acid methyl esters by the addition of 14% BF_3_ in methanol with hexane and transferred to a 95°C heating block for 1 h. Fatty acid methyl esters were then detected with a Varian 3900 gas chromatograph equipped with a DB-FFAP 15 m × 0.1 mm × 0.1 μm film thickness nitroterephthalic acid-modified polyethylene glycol capillary column (J&W Scientific from Agilent Technologies). Hydrogen was used as the carrier gas at a flow rate of 0.5 mL/min with a split ratio of 100:1. The inlet was heated to 250°C and the flame ionization detector was heated to 300°C with an air flow rate of 100 mL/min, hydrogen flow rate of 30 mL/min, and nitrogen make-up gas flow rate of 25 mL/min. The detector sampling frequency was set at 50 Hz. The initial oven temperature was 150°C with a hold for 0.25 min, followed by a 35°C/min ramp to 200°C, an 8°C ramp to 245°C, and a hold at 245°C for 15 min. Peaks were identified by comparison of retention times to an external standard mix (GLC-462, Nu-Chek Prep). The fatty acid data were expressed both qualitatively as relative weight percentage (wt%) of total fatty acids and quantitatively as concentrations (micrograms fatty acid per 100 microliters of whole blood).

#### Lipidomic analyses

Medio-level lipidomics (lipids with their individual fatty acyl species defined but not the specific location of the fatty acyl) were completed on the extracted lipids [27]. The organic lipid extracts were dried under N_2_ gas and reconstituted in 100 μL of the reconstitution solvent [65:35:5 acetonitrile/isopropanol/water (v/v/v) + 0.1% formic acid] for analysis by UHPLC-MS/MS using a Waters Acquity UHPLC system coupled to a Waters Synapt G2S*i* quadrupole time-of-flight mass spectrometer (QToF; Waters Corporation, Milford, MA, USA). UHPLC separation was completed using a binary multistep gradient described previously [28] with the Waters Acquity UHPLC charged surface hybrid (CSH) 1.7 μm × 2.1 mm × 150 mm column equipped with a VanGuard CSH 1.7 μm precolumn. Mobile phase A consisted of 60:40 acetonitrile/water (v/v) + 10 mM ammonium formate + 0.1% formic acid, and B was 90:10 isopropanol/acetonitrile (v/v) + 10 mM ammonium formate + 0.1% formic acid. The multistep gradient used was as follows: solvent B was 32% from 0 to 1.5 min, followed by a linear increase to 45% B from 1.5 to 4 min, 50% B from 4 to 8 min, 55% B from 8 to 18 min, 60% B from 18 to 20 min, 70% B from 20 to 35 min, 95% B from 35 to 40 min, 95% B from 40 to 45 min, a decrease to 32% B at 45.1 min, and a hold at 32% B until the 48 min mark. The flow was 250 μL/min, column compartment temperature was 45°C, autosampler temperature was 4°C, and the injection volume was 5 μL.

A retention time-based polarity-switching MS/MS method was used with operation in the negative electrospray ionization (ESI) mode (spray voltage −2.25 kV) from 0 to 27 min for polar lipid characterization (free fatty acids, lysophospholipids, phospholipids, sphingolipids) followed by positive ESI (spray voltage +2.25 kV) until the 45 min mark for nonpolar lipid characterization [triacylglycerols (TAGs) and cholesteryl esters (CEs)]. In both ion modes, the mass spectrometer was operated in high-resolution mode (continuum; ∼42,000 resolution), scan range *m/z* 100 to 1200, scan time 0.2 s/scan, cone voltage 40 V, cone gas flow 100 L/h, desolvation gas flow 600 L/h, nebulizer gas flow 7.0 bar, source temperature 140°C, and desolvation temperature 400°C. Spectra were lock mass-corrected using leucine enkephalin [*m/z* 554.2615 for (M−H)^−^ and *m/z* 556.2771 for (M+H)^+^]. MS/MS was performed under data-dependent acquisition (DDA) conditions for the top 5 ions with a ±1.0 Da isolation window and scan frequency 0.1 s/scan. Collision energies in the transfer cell were ramped from 30 V to 45 V at low mass (*m/z* 100) and 35 V to 60 V at high mass (*m/z* 1200) for negative ESI and from 30 V to 50 V at low mass (*m/z* 100) and 40 V to 60 V at high mass (*m/z* 1200) for positive ESI.

Preliminary lipid identifications were made using SimLipid software, which were then manually confirmed, and peak areas were determined using Progenesis QI. Lipid abundances were normalized using the internal standard belonging to the same lipid class as the analyte of interest. Semiquantitated concentration data are presented as mean ± SD of all analytes in nanomoles lipid per milliliter blood.

#### Statistical analyses

All analyses were performed using IBM SPSS Statistics Version 29.0.0.0. Stepwise multiple linear regression was used to iteratively determine blood biomarkers that predict dietary intakes of n–3 PUFA in all participants (*n* = 120). This process starts with zero predictors in the model and then adds predictors according to the largest significant gain in *R*^2^. As each predictor is added, the significance of each predictor’s B coefficient is checked to determine if the predictor stays in the model. This process resolves multicollinearity. Presently, stepwise modeling for dietary intake as the dependent variable was completed against *1*) lipidomic variables determined by UHPLC-MS/MS; *2*) all fatty acid data generated by GC-FID including sums and biomarker calculations; and *3*) individual fatty acids expressed qualitatively and quantitatively. This modeling was completed for the entire sample (*n* = 120), females only (*n* = 68), males only (*n* = 52), and individuals who did not consume fish oil supplements (*n* = 101). Two-tailed bivariate Pearson’s correlations were also performed between the dietary intake data and the lipidomic and fatty acid data, and independent *t* tests were used to compare the dietary intakes, lipidomic species, and fatty acids of females and males and those who reported fish oil supplement use and those who did not. Stepwise multiple linear regression was also used to determine lipidomic species that predicted commonly used fatty acid-based biomarkers. In the latter modeling, an additional cutoff of an *R*^2^ gain of ≥0.025 for each additional variable was applied to restrict the number of variables in the models. Statistical significance was generally inferred at *P* < 0.05.

## Results

The participants comprised 68 females and 52 males that did not differ in age (39.1 ± 12.0 y, mean ± SD) (Table 1). The males were significantly taller (180.9 ± 6.7 compared with 168.9 ± 6.5 cm), weighed more (80.1 ± 9.9 compared with 65.9 ± 10.2 kg), had a higher BMI (24.5 ± 2.7 compared with 23.1 ± 3.5 kg/m^2^), and had a higher energy intake (10.7 ± 2.6 MJ compared with 8.5 ± 1.8 MJ) than the females (Table 1). Although the mass intakes of the dietary components differed between males and females, there were no differences when intakes were expressed as energy percentage. There were 19 individuals (10 females, 9 males) who reported using fish oil supplements, and they had significantly higher intakes of EPA, DHA, and total n–3 intakes than those that did not report using supplements (Table 1). The supplement users were also older and consumed a higher percentage of their energy from protein.

**TABLE 1:** Participant characteristics and dietary intakes

Characteristic	All (*n* = 120)	Female (*n* = 68)	Male (*n* = 52)	No FO suppl (*n* = 101)	FO suppl (*n* = 19)
Age (y)	39.1 ± 12.0	40.0 ± 11.0	37.3 ± 13.0	37.9 ± 11.7	43.9 ± 12.2^1^
Height (cm)	174.1 ± 8.9	168.9 ± 6.5	180.9 ± 6.7^2^	1.7 ± 0.1	1.7 ± 0.1
Weight (kg)	72.0 ± 12.3	65.9 ± 10.2	80.1 ± 9.9^2^	73.9 ± 12.3	71.2 ± 12.8
BMI (kg/m^2^)	24.2 ± 3.2	23.1 ± 3.5	24.5 ± 2.7^2^	24.3 ± 3.1	23.7 ± 3.8
WB EPA+DHA (wt%)	4.4 ± 1.1	4.6 ± 1.0	4.2 ± 1.3	4.2 ± 1.0	5.4 ± 1.0^1^
Dietary intake					
FO suppl users (*n*)	19	10	9	0	19
Total energy (MJ)	9.4 ± 2.5	8.5 ± 1.8	10.7 ± 2.6^2^	9.5 ± 2.5	9.0 ± 2.3
Protein (g/d)	83 ± 24	76 ± 18	93 ± 27^2^	82.5 ± 23.3	88.5 ± 27.8
(energy %)	16.0 ± 2.5	15.9 ± 2.5	16.1 ± 2.6	14.6 ± 2.3	16.5 ± 3.2^1^
Carbohydrate (g/d)	238 ± 70	216 ± 53	266 ± 79^2^	240.5 ± 70.3	221.9 ± 66.0
(energy %)	45.1 ± 5.6	44.7 ± 5.3	45.6 ± 5.9	42.2 ± 5.7	40.9 ± 4.5
Fat (g/d)	90 ± 27	84 ± 22	98 ± 31^2^	90.7 ± 28.2	86.1 ± 20.4
(energy %)	37.1 ± 5.6	37.6 ± 5.0	36.6 ± 6.4	35.9 ± 5.8	36.4 ± 5.6
SFA (g/d)	33.6 ± 11.5	32.1 ± 10.2	35.5 ± 12.8	33.8 ± 11.8	32.2 ± 9.8
16:0	18.0 ± 5.9	16.9 ± 4.9	19.4 ± 6.8^2^	18.2 ± 6.1	16.9 ± 4.7
18:0	7.6 ± 2.8	7.0 ± 2.3	8.2 ± 3.3^2^	7.6 ± 2.9	7.4 ± 2.5
MUFA (g/d)	42.8 ± 13.5	39.2 ± 10.3	47.5 ± 15.7^2^	43.1 ± 14.0	40.9 ± 10.6
16:1n–7	1.5 ± 0.7	1.4 ± 0.5	1.7 ± 0.8^2^	1.5 ± 0.7	1.4 ± 0.5
18:1n–9	29.7 ± 10.1	27.1 ± 7.7	33.1 ± 11.8^2^	29.9 ± 10.5	28.6 ± 7.9
PUFA (g/d)	13.6 ± 4.6	12.5 ± 3.5	15.1 ± 5.5^2^	13.7 ± 4.8	12.1 ± 3.1
Total n–6 PUFA	11.2 ± 3.9	10.2 ± 3.0	12.4 ± 4.5^2^	13.7 ± 4.8	13.0 ± 3.1
18:2n–6	11.1 ± 3.8	10.2 ± 3.0	12.3 ± 4.5^2^	11.3 ± 4.0	10.1 ± 2.6
20:4n–6	0.12 ± 0.07	0.11 ± 0.06	0.14 ± 0.09	0.12 ± 0.08	0.13 ± 0.07
Total n–3 PUFA	2.2 ± 1.2	2.0 ± 0.7	2.4 ± 1.6	2.1 ± 1.2	2.7 ± 0.6^1^
18:3n–3	1.8 ± 1.0	1.9 ± 0.5	2.0 ± 1.5^2^	1.8 ± 1.1	1.6 ± 0.4
20:5n–3	0.17 ± 0.19	0.18 ± 0.20	0.15 ± 0.19	0.10 ± 0.12	0.51 ± 0.17^1^
22:5n–3	0.04 ± 0.04	0.04 ± 0.04	0.04 ± 0.04	0.04 ± 0.04	0.04 ± 0.04
22:6n–3	0.21 ± 0.21	0.22 ± 0.20	0.19 ± 0.22	0.16 ± 0.18	0.47 ± 0.19^1^

Values are mean ± SD.

Abbreviations: BMI, body mass index; DHA, docosahexaenoic acid; EPA, eicosapentaenoic acid; FO suppl, fish oil supplement; SD, standard deviation; SFA, saturated fatty acid; MUFA; monounsaturated fatty acid; PUFA, polyunsaturated fatty acid; WB, whole blood.

1 Significantly different than individuals who did not use fish oil supplements by independent *t* test, *P* < 0.05.

2 Significantly different than females by independent *t* test, *P* < 0.05.

For the lipidomic data, the 710 lipid species identified automatically were ranked by abundance and inspected manually. After the identifications were confirmed, semiquantitated concentrations were determined by manual calculations for 140 lipidomic acyl species. For the fatty acid data, 21 individual fatty acids that have been recommended to be reported in blood were determined qualitatively (wt% of total fatty acids) and quantitatively (micrograms fatty acid per 100 microliters of whole blood) [8]. The weight percentage of EPA + DHA in whole blood (Table 1) for the population was 4.4 ± 1.1 wt% and had a relatively normal distribution, with 2.1 wt% being the lowest value and 8.0 wt% (an individual consuming fish oil supplements) being the highest value in the present population.

The stepwise linear regression models for predicting intake of EPA + DHA in the sample and subpopulations are presented in Table 2, which indicates the constant, the variables included in each model, the B coefficients and their standard error and *P* value along with the *R*^2^ for the model. This information can be used to define the intake of EPA + DHA of a sample. As an example, for the entire sample population of 120 participants, EPA + DHA intake can be estimated from the blood levels of lipid species of ceramide phosphoethanolamines (Cer), cholesteryl esters (CE), plasmenyl phosphatidylethanolamines (PE P), phosphatidylserines (PS) and triacylglycerols (TAG) such that EPA + DHA intake (g/d) = 0.590 + (0.061) [PE P -16:0_20:5] + (−0.006) [PE P-18:0_22:4] + (−0.130) [TAG 14:0_16:0_16:0] + (.001) [CE 22:6] + (−0.254) [Cerd14:2_23:0] + (0.008) [PS 18:0_22:6] with a moderately strong *R*^2^ of 0.52 (Table 2). The zero-order (Pearson) correlations confirmed that PE P-16:0_20:5 was the individual variable with the strongest correlation to EPA + DHA intake (*r* = 0.59, *P* < 0.00001) in the sample population (Supplemental Table 1). When the sexes were separated, the *R*^2^ values of these lipidomic-based models increased to 0.62 for females (*n* = 68) and to 0.72 for males (*n* = 52), with no overlap in the predictive variables selected for the sex-based models. The model for females included CE species, phosphatidylethanolamine (PE) and PE P species containing DHA, and PE P containing adrenic acid (22:4n–6), whereas the model for males contained PE and PE P species containing EPA, a TAG, and a lysophosphatidylethanolamine. The *R*^2^ value decreased to 0.33 when only the participants who did not consume fish oil supplements (*n* = 101) were included (*R*^2^ = 0.49 in females only and 0.63 in males only, data not shown). Interestingly, PE P-16:0_22:6 was the first predictive variable instead of PE P-16:0_20:5 in the model for the entire sample (*n* = 120). Individuals who did not consume fish oil had lower fatty acid levels (GC-FID) of EPA + DHA in their blood (Table 1), with EPA being 37% lower and DHA being 17% lower than that of users of fish oil (data not shown).

**TABLE 2:** Stepwise linear regression models for predicting dietary EPA + DHA intakes from lipidomic species measured in whole blood

Variables	B	SE	*P*	Model *R*^2^
All participants (*n* =120)				
(Constant)	0.590	0.239	0.015	0.52
PE P-16:0_20:5	0.061	0.015	<0.001	
PE P-18:0_22:4	–0.006	0.003	0.045	
TAG 14:0_16:0_16:0	–0.130	0.036	<0.001	
CE 22:6	0.001	0.000	0.002	
Cer-PE d14:2_23:0	–0.254	0.088	0.005	
PS 18:0_22:6	0.008	0.003	0.022	
Females only (*n* = 68)				
(Constant)	0.263	0.263	0.322	0.62
CE 20:5	0.001	0.000	<0.001	
PE P-18:1_22:6	0.036	0.017	0.038	
PE P-18:0_22:4	–0.014	0.004	0.002	
PE 16:0_22:6	0.029	0.010	0.005	
CE 18:1	–0.002	0.001	0.029	
Males only (*n* = 52)				
(Constant)	0.195	0.132	0.147	0.72
PE P-16:0_20:5	0.217	0.022	<0.001	
PE 18:1_20:5	–0.435	0.072	<0.001	
TAG 16:0_16:0_18:1	–0.017	0.004	<0.001	
LPE 16:0	0.496	0.124	<0.001	
No FO supplements (*n* = 101)				
(Constant)	0.149	0.165	0.370	0.33
PE P-16:0_22:6	0.047	0.008	<0.001	
PE 18:0_20:4	–0.010	0.003	0.002	
TAG 14:0_16:0_16:0	–0.064	0.030	0.034	

Abbreviations: CE, cholesteryl ester; Cer-PE, ceramide phosphoethanolamine; DHA, docosahexaenoic acid; EPA, eicosapentaenoic acid; FO, fish oil; LPE, lysophosphatidylethanolamine; PE, phosphatidylethanolamine; PE P, plasmenyl phosphatidylethanolamine; PS, phosphatidylserine, SE, standard error; TAG, triacylglycerol.

For fatty acid GC-FID based variables, modeling with all fatty acid variables including calculations were completed (Table 3) followed by modeling with individual fatty acids expressed qualitatively (wt%) and quantitatively (micrograms fatty acid per 100 μL) (Table 4). When compared with the models with lipidomic variables, the fatty acid-based models for predicting EPA + DHA intakes were weaker in the entire sample, with *R*^2^ values ranging from 0.36 to 0.40 compared with 0.52 when lipidomic variables were used. The fatty acid-based models were also slightly weaker for females only (*R*^2^ values 0.42–0.48 compared with 0.62) and males only (*R*^2^ values 0.44–0.50 compared with 0.72) analyses. In all individuals not using supplements (*n* = 101), the fatty acid-based models were similar to (*R*^2^ = 0.30 for individual fatty acids as concentrations) and slightly stronger (*R*^2^ = 0.38 for individual fatty acids as percentages, *R*^2^ = 0.40 for all fatty acid variables) than the lipidomic-based model (*R*^2^ = 0.33). When the individuals not using supplements were separated by sex (data not shown), the fatty acid models were slightly weaker for females (*R*^2^ values 0.32–0.40) and similar for males (*R*^2^ values 0.59–0.61) compared with the lipidomic models for females (*R*^2^ = 0.49) and males (*R*^2^ = 0.63). For all fatty acid variables including calculations, the percentage n–3 highly unsaturated fatty acid (HUFA) [(20:3n–3 + 20:5n–3 + 22:5n–3 + 22:6n–3) / (20:3n–6 + 20:4n–6 + 22:4n–6 + 22:5n–6 + 20:3n–3 + 20:5n–3 + 22:5n–3 + 22:6n–3) × 100] was the only predictive variable in the models for all participants and females only, and it was the first predictive variable for the models of males only and for those that did not use fish oil supplements. The zero-order (Pearson) correlations for all participants confirmed that the percentage n–3 HUFA (*r* = 0.63, *P* < 0.00001) had the strongest association with EPA + DHA intake, although EPA + DHA wt% (*r* = 0.57, *P* < 0.00001), the sum of total n–3 PUFA wt% (*r* = 0.57, *P* < 0.00001), and the ratio of n–6 to n–3 PUFA (*r* = −0.54, *P* < 0.00001) were of relatively similar strength (Supplemental Table 2).

**TABLE 3:** Stepwise linear regression models for predicting dietary EPA + DHA intakes using all fatty acids variables including calculations measured in whole blood

Variables	B	SE	*P*	Model *R*^2^
All participants (*n* = 120)				
(Constant)	–1.049	0.165	<0.001	0.40
% n–3 HUFA	0.045	0.005	<0.001	
Females only (*n* = 68)				
(Constant)	–1.330	0.223	<0.001	0.48
% n–3 HUFA	0.054	0.007	<0.001	
Males only (*n* = 52)				
(Constant)	–0.210	0.287	0.468	0.44
% n–3 HUFA	0.040	0.007	<0.001	
23:0 wt%	–3.256	0.995	0.002	
No FO suppl (*n* = 101)				
(Constant)	–0.864	0.213	<0.001	0.40
% n–3 HUFA	0.031	0.005	<0.001	
18:0 conc	0.027	0.007	<0.001	
14:1 conc	–2.996	1.086	0.007	
22:0 conc	–0.193	0.083	0.022	

Abbreviations: % n–3 HUFA (20:3n–3 + 20:5n–3 + 22:5n–3 + 22:6n–3) / (20:3n–6 + 20:4n–6 + 22:4n–6 + 22:5n–6 + 20:3n–3 + 20:5n–3 + 22:5n–3 + 22:6n–3) × 100; conc, μg fatty acid/100 μL of whole blood; DHA, docosahexaenoic acid; EPA, eicosapentaenoic acid; FO suppl, fish oil supplement; SE, standard error; wt%, relative % of total fatty acids.

**TABLE 4:** Stepwise linear regression models for predicting dietary EPA + DHA intakes using qualitative and quantitative individual fatty acids measured in whole blood

Intake	Variables	B	SE	*P*	Model*R*^2^
Individual FA wt%					
All participants(*n* = 120)	(Constant)	0.053	0.215	0.806	0.36
	22:6n–3 wt%	0.212	0.042	<0.001	
	22:5n–6 wt%	–2.113	0.560	<0.001	
Females only(*n* = 68)	(Constant)	–2.101	0.388	<0.001	0.47
	22:6n–3 wt%	0.389	0.054	<0.001	
	18:2n–6 wt%	0.057	0.016	<0.001	
Males only(*n* = 52)	(Constant)	0.135	0.534	0.801	0.49
	22:5n–6 wt%	–3.639	0.745	<0.001	
	23:0 wt%	–3.351	0.978	0.001	
	22:5n–3 wt%	0.670	0.191	<0.001	
	18:0 wt%	0.063	0.030	0.041	
No FO suppl(*n* = 101)	(Constant)	–1.438	0.441	0.002	0.38
	22:6n–3 wt%	0.082	0.041	0.052	
	22:4n–6 wt%	–0.254	0.115	0.029	
	18:0 wt%	0.073	0.021	<0.001	
	18:1n–7 wt%	0.428	0.158	0.008	
	20:5n–3 wt%	0.188	0.085	0.030	
Individual FA conc					
All participants(*n* = 120)	(Constant)	0.275	0.200	0.171	0.38
	22:6n–3 conc	0.114	0.020	<0.001	
	22:5n–6 conc	–1.040	0.218	<0.001	
	23:0 conc	–0.594	0.290	0.043	
Females only(*n* = 68)	(Constant)	0.320	0.141	0.027	0.42
	20:5n–3 conc	0.225	0.043	<0.001	
	20:3n–9 conc	–1.938	0.487	<0.001	
Males only(*n* = 52)	(Constant)	0.300	0.312	0.341	0.50
	22:5n–6 conc	–1.710	0.342	<0.001	
	23:0 conc	–1.543	0.418	<0.001	
	22:5n–3 conc	0.324	0.091	<0.001	
	18:0 conc	0.021	0.009	0.019	
No FO suppl(*n* = 101)	(Constant)	–0.044	0.145	0.761	0.30
	22:6n–3 conc	0.077	0.015	<0.001	
	22:5n–6 conc	–0.563	0.181	0.002	

Abbreviations: wt%, relative % of total fatty acids; conc, μg fatty acid/100 μL of whole blood; DHA, docosahexaenoic acid; EPA, eicosapentaenoic acid; FA, fatty acid; FO suppl, fish oil supplement; SE, standard error.

Lipidomic and fatty acid-based biomarker variables were also modeled against the intakes of α-linolenic acid (ALA), total n–3 PUFA, and EPA and DHA individually (Supplemental Tables 3–10). The *R*^2^ values of the models were consistently higher for EPA intake and DHA intake compared with ALA and total n–3 PUFA intakes. The pattern of increased *R*^2^ values when modeling females only and males only, particularly with lipidomic variables that was observed with EPA + DHA intakes, was observed across ALA (all participants = 0.11 compared with females = 0.25 and males = 0.35, *R*^2^), total n–3 PUFA (all participants = 0.13 compared with females = 0.48 and males = 0.24, *R*^2^), EPA (all = 0.48 compared with females = 0.69 and males = 0.76, *R*^2^), and DHA (all = 0.47 compared with females = 0.59 and males = 0.71, *R*^2^). The plasmalogen PE P-16:0_20:5 commonly appeared as the first lipidomic variable entered into several of the models including: all participants for total n–3 PUFA intakes; all participants, females only, and males only for dietary EPA; and males only for DHA intakes. For DHA intakes, PE P-16:0_22:6 (all participants), PE P-18:1_22:6 (females only) and PE P-18:0_22:4 (all and females) plasmalogens were selected as either the first or second variable.

It is not surprising that sex differences were observed in the fatty acid compositional analyses by GC-FID (Supplemental Table 11) and in the lipidomic analyses by UHPLC–MS/MS (Supplemental Table 12). For the fatty acids, females had lower wt% of 18:1n–9, 18:3n–6, 22:5n–3 and total monounsaturates and higher percentages of 24:1n–9, 18:2n–6, 22:6n–3, and total n–6 PUFA compared with males. For the lipidomics, sex differences were observed in 15 glycerophosphocholines (11 of which were higher in females), 9 glycerophosphoethanolamines (7 of which were higher in males), 4 phosphosphingolipids (3 of which were higher in females), and 4 glycerolipids (which were higher in males). The glycerophosphocholines that were higher in females all contained some form of ≥1 unsaturated fatty acyl. Concentrations of the remaining acyl species that were determined but were not different by sex included 21 glycerophosphocholines (Supplemental Table 13), 29 glycerophosphoethanolamines (Supplemental Table 14), 24 other polar lipids (Supplemental Table 15), and 30 nonpolar lipids (Supplemental Table 16).

Stepwise linear regression modeling was also used to examine models of lipidomic species that best predicted the commonly used and established fatty acid-based blood biomarkers (Table 5). The model *R*^2^ values ranged from 0.84 to 0.91 when the modeling was stopped (when the addition of the next variable resulted in <0.025 *R*^2^ gain). Once again, various PE plasmalogen species were identified as predictive variables. PE P-18:0_20:5 was associated with EPA wt%, PE P-18:0_22:6 with DHA wt%, and PE P-18:0_22:6 and PE P-18:1_20:5 were associated with EPA + DHA wt%. PE P-18:0_20:5 was also associated with the percentage of n–3 HUFA along with a phosphatidylcholine (PC) species, PC 18:0_22:6. PE P-18:1_20:5 and PC 18:0_22:6 were both associated with total n–3 PUFA wt% and the n–6/n–3 ratio. PC 18:2_20:5 was also associated with total n–3 PUFA wt % and PE P-18:0_22:6 with the n–6/n–3 ratio. Species containing n–6 PUFA were also found to be inversely correlated with the fatty acid biomarkers of n–3 PUFA status and included PE P-16:0_22:5 for DHA wt%, PE P-16:0_20:4 for EPA + DHA wt%, and PE P-18:0_22:4 for the percentage of n–3 HUFA. Highly abundant PC species containing either EPA or DHA were also predictive of the n–3 blood biomarker status along with opposite associations with species containing either 20:4 (EPA wt% and percentage n–3 HUFA) or 18:2 (n–6/n–3 ratio). Interestingly, PC 18:1_18:1 was inversely associated with n–3 PUFA status in all the biomarkers except for percentage n–3 HUFA.

**TABLE 5:** Stepwise linear regression models^1^ for determining explanatory lipidomic species variables for common fatty acid-based n–3 biomarkers as measured in whole blood

FA n–3 biomarkers	Variables	B	SE	*P*	Model *R*^2^
EPA wt%	(Constant)	0.46	0.082	<0.001	0.90
	PC 18:0_20:5	0.094	0.004	<0.001	
	PC 16:0_20:4	–0.007	0.001	<0.001	
	PC 18:1_18:1	–0.004	0.001	<0.001	
	PE P-18:0_20:5	0.039	0.005	<0.001	
DHA wt%	(Constant)	3.699	0.256	<0.001	0.87
	PC 18:0_22:6	0.052	0.006	<0.001	
	PC 18:1_18:1	–0.009	0.001	<0.001	
	PE P-18:1_22:6	0.197	0.018	<0.001	
	PE P-16:0_22:5	–0.025	0.004	<0.001	
	TAG 18:0_18:1_18:1	–0.016	0.003	<0.001	
EPA + DHA wt%	(Constant)	3.994	0.363	<0.001	0.85
	PC 18:0_22:6	0.100	0.009	<0.001	
	PC 18:1_18:1	–0.013	0.002	<0.001	
	PE P-18:1_20:5	0.218	0.028	<0.001	
	PE P-16:0_20:4	–0.023	0.004	<0.001	
% n–3 HUFA	(Constant)	34.631	1.371	<0.001	0.91
	PE P-18:0_20:5	0.884	0.056	<0.001	
	PE P-18:0_22:4	–0.305	0.026	<0.001	
	PC 18:0_22:6	0.351	0.039	<0.001	
	PC 18:0_20:4	–0.13	0.015	<0.001	
Total n–3 PUFA wt%	(Constant)	7.002	0.398	<0.001	0.87
	PE P-18:1_20:5	0.353	0.035	<0.001	
	PE P-16:0_22:5	–0.029	0.006	<0.001	
	PC 18:2_20:5	0.244	0.103	0.02	
	PC 18:1_18:1	–0.011	0.002	<0.001	
	PC 18:0_22:6	0.072	0.012	<0.001	
	TAG 16:0_18:1_18:1	–0.011	0.002	<0.001	
n–6/n–3 ratio	(Constant)	5.078	0.426	<0.001	0.84
	PC 18:0_22:6	–0.101	0.011	<0.001	
	PC 18:1_18:1	0.016	0.002	<0.001	
	PE P-18:1_20:5	–0.209	0.033	<0.001	
	PC 16:1_22:6	–0.226	0.038	<0.001	
	PE P-18:0_22:6	–0.05	0.007	<0.001	
	PC 16:1_18:2	–0.036	0.008	<0.001	

Abbreviations: % n–3 HUFA, (20:3n–3 + 20:5n–3 + 22:5n–3 + 22:6n–3) / (20:3n–6 + 20:4n–6 + 22:4n–6 + 22:5n–6 + 20:3n–3 + 20:5n–3 + 22:5n–3 + 22:6n–3) × 100; wt%, relative % of total fatty acids; DHA, docosahexaenoic acid; EPA, eicosapentaenoic acid; FA, fatty acid; PC, phosphatidylcholine; PE P, plasmenyl phosphatidylethanolamine; PUFA, polyunsaturated fatty acid; TAG, triacylglycerol.

1 Models were stopped when the *R*^2^ gain was <0.025.

## Discussion

Lipidomic and fatty acid analyses of whole blood from 120 Danish participants were completed and examined for their ability to predict intakes of n–3 PUFA as determined by a web-based 7-d food diary [21]. The lipidomic analyses tentatively identified 710 lipids at the medio level (fatty acyl species) from which 140 identities of the most highly abundant lipids were confirmed and semiquantitated manually. Stepwise linear regression indicated that lipidomic species could predict dietary EPA + DHA intake slightly better than GC-FID fatty acids in the entire sample (*R*^2^ = 0.52 compared with 0.40, respectively) but slightly worse when supplement users were excluded (*R*^2^ = 0.33 compared with 0.40, respectively). However, the stepwise models gained strength when females and men were examined separately, particularly with lipidomics, as sex differences were observed for 32 individual lipid species and only 8 fatty acids. Stepwise linear regression also indicated that lipidomic species can predict and reflect commonly used GC-FID fatty acid-based n–3 PUFA blood biomarkers of EPA wt% (*R*^2^ = 0.90), DHA wt% (*R*^2^ = 0.87), EPA + DHA wt% (*R*^2^ = 0.85), percentage n–3 HUFA (*R*^2^ = 0.91), and the n–6/n–3 ratio (*R*^2^ = 0.84). This should help with the interpretation of lipidomic analyses as this approach can enable translation across GC-FID and lipidomic-based data.

PE P-16:0_20:5 was the first explanatory lipidomic variable selected by stepwise regression for modeling of EPA + DHA intakes in all participants and in males only. In females only, CE 20:5 was the first variable selected followed by PE P-18:1_22:6, whereas in those not using supplements, PE P-16:0_22:6 was the first explanatory variable selected. These lipidomic models also contained an n–6 LCPUFA-containing lipid (PE P-18:0_22:4 or PE 18:0_20:4) except for males only, and a TAG species with 18-carbon or less saturated or monounsaturated fatty acyls except for the females only. The inclusion of these n–6 LCPUFA and TAG lipids in the stepwise models were based on significant inverse relationships with EPA + DHA dietary intakes that were not colinear with the positive associations of the EPA- and DHA-containing plasmalogens. The inverse relationships with n–6 LCPUFA-containing lipids are expected given previous observations in the literature based on Lands’ cycle remodeling of glycerophospholipids [11,29]. The inverse relationship with a saturated/monounsaturated TAG molecule is likely related to the hypotriglyceridemic effect of n–3 LCPUFA on circulating lipoproteins [30]. The lack of a TAG variable in the females only model may be related to a previous observation that fish oil has greater TAG-lowering effect in males than in females [31]. Instead of a TAG, CE 20:5 and CE 18:1 were predictors of EPA + DHA intakes in the females only model. Although there is some evidence of females having increased DHA but not necessarily EPA levels in the CE fraction compared with males [32], it is likely these differences in TAG and CE variables in the models of females and males are related to the well-documented sex differences in lipoprotein metabolism [33].

Individuals with the highest n–3 LCPUFA intakes and blood levels also consumed supplements [22,34]. As blood levels of n–3 LCPUFA increase, the proportion of EPA to DHA increases [20], and 20:5n–3 increases and decreases rapidly in blood compared with 22:6n–3 in response to dietary intake, especially in the erythrocyte fraction [35]. In the present study, the blood levels of 20:5n–3 + 22:6n–3 were ∼30% higher in the fish oil supplement consumers than in those who did not consume fish oil supplements. In those that did not consume fish oil supplements, PE P-16:0_22:6 replaced PE P-16:0_20:5 as the first explanatory variable in the lipidomic variable model. This shift toward increasing strength of 22:6n–3 as a predictor of intake in individuals with lower n–3 LCPUFA status and increasing strength of 20:5n–3 as a predictor of intake in individuals with higher n–3 LCPUFA status may also be caused by the frequency of n–3 LCPUFA intake.

Plasmalogens were repeatedly identified as strong n–3 PUFA intake biomarkers in whole blood in the various models generated in this study. Plasmalogen levels in plasma (∼0.6% of total phospholipids) are much lower than erythrocyte levels (∼9.2% of total phospholipids) [36]; therefore, the PE plasmalogen associations we observed in this study examining whole blood are likely being dictated by levels in the erythrocytes. However, increases in human plasma EPA- and DHA-containing plasmalogens with increased EPA and DHA intakes have been observed. Plasma PE P-16:0_20:5, PE P-18:0_20:5, PC P-16:0_20:5, PE P-16:0_22:6, PE P-18:0_22:6, PE P-18:1_22:6, and PC P-16:0_22:6 increase after supplementation with krill oil capsules but not fish oil for 30 d [37]. Plasma PE P-16:0_22:6, PE P-18:0_22:6, and PE P-18:1_22:6 also increase after supplementation with DHA-containing microalgae oil as a powder incorporated into food items for 10 wk [38]. The present findings may be restricted to the analysis of whole blood with the possibility of extrapolation to erythrocyte analysis but not plasma; however, further research examining these specific blood fractions appears to be necessary.

It is well documented that fatty acid blood data by GC-FID reflects dietary intake of EPA + DHA [6,16]. When the Lands equation for predicting HUFA compositions in human blood from dietary intakes of fatty acids was validated using 92 study groups from 34 separate studies (23 intervention studies, 11 observational studies), the *R*^2^ value of the relationship was 0.53 [39]. Intervention studies enable a much more precise estimate of dietary intake of EPA + DHA. Previously, in a small invasive fish oil dose-response study of highly compliant participants (as confirmed by weekly dried blood spot analyses) designed specifically to examine the relationship between the dietary intake of EPA + DHA to whole blood levels of EPA + DHA, the predictive equation resulted in an *R*^2^ of 0.94 [16]. Therefore, the *R*^2^ values generated in the present study from web-based dietary assessment data of free-living individuals should be considered quite strong. It was a bit surprising that the *R*^2^ from the lipidomic variables was only slightly stronger than the *R*^2^ from the fatty acid variables when the entire sample was examined and slightly weaker when supplement users were excluded, although this appears to be caused by more sex-based differences in the lipidomic variables.

In the present study, the *R*^2^ values of predictive models using quantitative individual fatty acids were relatively similar to those using qualitative individual fatty acids, which was not expected. There is a preference for expressing fatty acid blood data as relative percentage [20] because relative percentage data better highlights the metabolic competition between the fatty acids for placement in complex lipids and the variation in the data is greatly reduced [8]. When all fatty acid variables including calculations were modeled, the percentage n–3 HUFA was the main stepwise variable for predicting EPA + DHA. The percentage n–3 HUFA is a composite score that includes all the n–3 and n–6 HUFAs (≥20 carbons, ≥3 carbon=carbon double bonds). Hence, it is logical that this would be the most predictive variable and no other HUFA would be included in the model due to collinearity. Although percentage n–3 HUFA has been demonstrated to be a very useful source for assessing EPA and DHA status of an individual [29,40], the sum of the percentages of EPA and DHA is used more often due to the popularity of the Omega-3 Index [15,20].

The examination of lipidomic species that can predict commonly used GC-FID-based n–3 PUFA biomarkers allows a unique perspective on how to interpret lipidomic data for comparison to historical fatty acid-based analyses and research. However, the comparison of GC-FID and lipidomic biomarkers appears to be limited to one other article [41]. This previous report identified PC 16:0_20:5 and PC 16:0_22:6 as lipidomic surrogate biomarkers of the Omega-3 Index, whereas our stepwise model for EPA + DHA wt% in whole blood included PC 18:0_22:6 and PE P-18:1_20:5 as positive predictors and PC 18:1_18:1 and PE P-16:0_20:4 as negative predictors. The previous model used serum/plasma-based analyses of participants in intervention studies (categorical design) using relatively high doses of supplements, specifically 3g/d EPA, 3g/d DHA, and/or high-dose fish oil (3g EPA + 2 g DHA/d) in comparison with sunflower or olive oil controls but without dietary assessments [41]. PCs in lipoprotein monolayers are the dominant phospholipid in serum/plasma [42,43] and could have influenced the identification of PC 16:0_20:5 and PC 16:0_22:6 previously, and we have seen in the present study that EPA + DHA from traditional food compared with EPA + DHA from supplements may influence blood lipidomics.

Previously, the population of Denmark has been categorized as a country with one of the highest consumptions (≥550 mg EPA + DHA)/d) [44] and blood levels of EPA + DHA (>8 wt% EPA + DHA erythrocyte equivalents) [20] in the world. However, in the present study sample, intake estimates were 375 ± 398 mg/d and whole blood EPA+DHA was 4.41 ± 1.13 wt%, which corresponds to erythrocyte equivalents closer to 6 wt%. Dietary assessments of short duration such as food records and recalls can misrepresent EPA + DHA intakes in individuals who consume fish sporadically [5] such that the determination of “usual” intakes of fish may also require an additional food propensity questionnaire to capture intake over longer periods of time [45]. However, based on aggregate data in recent years, there is evidence of a slight decrease of fish in the food supply in Denmark that may partially explain the lower intakes and blood values observed presently compared to older reports in the literature. The FAO of the United Nations estimated the crude fish food supply of 18.5 kg per capita in 2015 decreased to 15.7 kg per capita in 2021 [46] although Euromonitor International estimated fish and seafood buying of 8.3 kg per capita in 2018 and 7.2 kg per capita in 2022 [47].

There are methodological limitations with this exploratory study. The lipidomic analyses used were discovery and untargeted based (discussed in the next paragraph), and a data mining statistical analysis was used. Stepwise regression is often criticized as it requires no forethought and theoretical input about the variables entered into the model, resulting in meaningless output [48]. In addition, Type I error is inflated due to the number of predictors that are examined, *P* values can be misleadingly small, and a focus on *R*^2^ can result in overly complex models [48,49]. In our study, splitting the participants by sex resulted in a considerable *R*^2^ gain and completely different predictive variables compared to when all participants were examined together, but this also resulted in a much smaller sample size (for example, *n* = 68 and *n* = 52 compared with *n* = 120) and leads to less confidence in the predictive models. As a result, there needs to be considerable precaution when interpreting the models generated for females only and males only, but sex differences definitely need to be considered when examining lipidomic blood data in the future. The fact that stepwise regression generates output from just the data may be an advantage as it eliminates preconceived biases about the variables. For example, PC 16:0_22:6 was identified as a main lipidomic biomarker of n–3 PUFA using a hierarchical approach in a previous study [41] as discussed above. Although PC 16:0_22:6 was significantly correlated to EPA + DHA intakes in our study (*r* = 0.40, *P* < 0.00001) (Supplemental Table 1), it did not appear in any of our stepwise models. There is evidence of metabolic adaptations to maintain plasma levels of PC 16:0_22:6 in females when intakes of n–3 PUFA are low, especially during pregnancy [50,51]. Because PC 16:0_22:6 levels are protected and maintained at a minimum level, it will not be able to discriminate n–3 PUFA status at lower levels of intakes.

The lipidomic workflow used herein should be considered when interpreting the lipids identified. The UHPLC-MS/MS method used scheduled (retention time-based) ESI polarity switching [[52], [53], [54], [55]] for untargeted lipidomic analyses of whole blood within a single analytical run instead of alternating positive and negative scans [[56], [57], [58], [59], [60]]. Although platforms capable of fast switching do exist [61,62], the Waters Synapt G2S*i* QToF has a switching dwell time of ∼20 s that is not fast enough for UHPLC-based protocols. As a result, monoacylglycerols, diacylglycerols, free cholesterol and cardiolipins were not measured in the present workflow because they eluted when the ESI polarity was in a mode that limited their ionization and, thus, prevented their detection. In addition, the top 5 DDA approach used herein biases the identification toward highly abundant lipids over those of low abundance. Although the present method does not characterize the full lipidome, it is well suited for untargeted discovery examinations of highly abundant acyl species of samples or what we have termed “macrolipidomic” analysis in the past [27]. In addition, the lipids were identified at the “medio” level [27] where the carbon number and level of unsaturation of the fatty acid constituents were determined for each molecule, but regional, positional and geometric isomers were not determined. Although prior knowledge about fatty and lipid metabolism allows fatty acyls identified as 20:4 and 22:6 to be interpreted as 20:4n–6 and 22:6n–3, respectively, there are fatty acyl isomers such as 18:3 (18:3n–3 compared with 18:3n–6) and 22:5 (22:5n–3 compared with 22:5n–6) that can coexist in blood [63] and therefore result in difficulties in interpretation. Methods of characterizing double-bond locations that provide a deeper level of structural information (i.e., “infinio”) about lipids would help nutritional interpretation of lipidomic data [[64], [65], [66], [67]]. Significant progress has recently been made in analytical technologies and the coverage and expansion of *in silico* lipidomic databases for discovery/untargeted research [68]. However, the widespread adoption of these approaches remains a challenge due to the costly nature of the required infrastructure and the lack of standardization within the lipidomics community [69]. Full/accurate quantitation using isotope dilution mass spectrometry and the inclusive analysis of lipid species of low abundance such as monoacylglycerols, diacylglycerols, cardiolipins, and oxylipins could also provide additional insights about the relationship between the dietary intake and blood levels of LCPUFA.

In conclusion, to our knowledge, this is the largest examination of potential biomarkers of n–3 PUFA intake using and comparing lipidomic and fatty acid-based analyses. It was surprising that models for predicting dietary intakes of EPA + DHA by lipidomic and fatty acid-based data were of relatively similar strength when the entire sample was examined. Therefore, despite providing less molecular information, the more economical GC-FID-based analyses still have an important role as a research tool for fatty acid and lipid nutrition. However, we have also shown that this may be a result of a greater effect of sex on lipidomic variables because lipidomic biomarkers retain lipid class information. Lipidomic molecules such as PE P-16:0_20:5, PE P-16:0_22:6, and PE P-18:1_22:6 in whole blood appear to provide similar insights about dietary intakes of EPA + DHA, but these observations need to be confirmed in other studies and populations, especially given the exploratory design and analysis of the present study. We also defined potential lipidomic models that can reflect commonly used fatty acid-based biomarkers such as EPA + DHA, the percentage of n–3 HUFA, and the n–6/n–3 ratio. These insights will allow lipidomic data to be interpreted similarly and compared with previous studies using GC-FID fatty acid data. Understanding how to translate between fatty acid and lipidomic data is critical for the embrace of lipidomics as a nutritional research approach.

## Author contributions

The authors’ responsibilities were as follows – APB-J: served as the principal investigator for participant recruitment, sample collection, and the dietary intake determinations; APB-J, TC: were involved in the dietary assessment study design, data collection, and data processing; KDS: served as the principal investigator for the determination and examination of fatty acid and blood biomarkers; KDS, JJA-H: were involved in the biomarker study design and the initial draft of the manuscript; JJA-H: completed the fatty acid and lipidomic analyses of whole blood; KDS: completed the statistical analyses; and all authors: read, reviewed, and approved the final manuscript.

### Conflict of interest

Ken D Stark is an Editorial Board Member for *The Journal of Nutrition* and played no role in the Journal’s evaluation of the manuscript and is currently on the Board of Directors of the International Society for the Study of Fatty Acids and Lipids. JJA-H completed this research at the University of Waterloo during his PhD but currently works for BPGbio Inc., a company that provides lipidomic analyses. All other authors report no conflicts of interest.

### Funding

The fatty acid and lipidomic analyses were supported by an operating grant from the Natural Sciences and Engineering Research Council of Canada (#RGPIN-2018-04334) to KDS; JJA-H was supported by an Natural Sciences and Engineering Research Council of Canada Doctoral Scholarship and an Ontario Graduate Scholarship. KDS also received salary support from the Canada Research Chairs program as a Chair in Nutritional Lipidomics (950–228125). The participant recruitment, sample collection and dietary assessment research received no specific grant from any funding agency, commercial or not-for-profit sectors.

## Data availability

Data described in the manuscript is restricted in accordance with Danish law, such that the personal individual dietary intake data, fatty acid and lipidomic blood levels presented in this study can only be accessed through the servers at The Technical University of Denmark. Access is granted upon request if the applicant fulfills defined criteria. DTU Food can be contacted by e-mail: apbj@food.dtu.dk.
