# Supplementary material for: Lipidomic and Fatty Acid Biomarkers in Whole Blood Can Predict the Dietary Intake of Eicosapentaenoic and Docosahexaenoic Acids in a Danish Population
Source: J Nutr. 2024 May 4;154(7):2108–19. doi: 10.1016/j.tjnut.2024.04.038 (PMC11282468; doi:10.1016/j.tjnut.2024.04.038)
Supplement: Multimedia component 1 [file mmc1.docx]

**Supplementary Table 1.** Pearson’s correlations of whole blood acyl lipid species (nmol/mL) associated with intakes of eicosapentaenoic acid (EPA) plus docosahexaenoic acid (DHA) (g/d)

|  | EPA + DHA Intake | |
| --- | --- | --- |
| Medio lipid | r | P-value |
| PE P-16:0_20:5 | 0.59 | < 0.00001 |
| PE P-18:1_20:5 | 0.58 | < 0.00001 |
| PE P-18:0_20:5 | 0.55 | < 0.00001 |
| PE 16:0_20:5 | 0.53 | < 0.00001 |
| PE P-16:0_22:6 | 0.51 | < 0.00001 |
| FFA 20:5 | 0.50 | < 0.00001 |
| PE P-18:1_22:6 | 0.50 | < 0.00001 |
| PS 18:0_20:5 | 0.50 | < 0.00001 |
| PC 18:0_22:6 | 0.48 | < 0.00001 |
| PS 16:0_20:5 | 0.48 | < 0.00001 |
| CE 20:5 | 0.46 | < 0.00001 |
| PE P-18:0_22:6 | 0.46 | < 0.00001 |
| CE 22:6 | 0.43 | < 0.00001 |
| PE P-20:0_22:6 | 0.43 | < 0.00001 |
| PC 18:0_20:5 | 0.42 | < 0.00001 |
| PI 18:0_20:5 | 0.42 | < 0.00001 |
| PC 16:0_22:6 | 0.40 | < 0.00001 |
| PE 18:1_20:5 | 0.39 | < 0.00001 |
| LPE 20:5 | 0.39 | < 0.0001 |
| PE 18:1_22:6 | 0.38 | < 0.0001 |
| PE P-18:0_22:4 | -0.38 | < 0.0001 |
| LPC 20:5 | 0.36 | < 0.0001 |
| PI 16:0_20:5 | 0.34 | < 0.001 |
| PS 18:0_22:6 | 0.33 | < 0.001 |
| PE 16:0_22:6 | 0.32 | < 0.001 |
| PC 14:0_20:5 | 0.31 | < 0.001 |
| PC 16:0_20:5 | 0.31 | < 0.001 |
| PC 16:1_22:6 | 0.31 | < 0.001 |
| PC 18:1_22:6 | 0.31 | < 0.001 |
| PC 18:2_20:5 | 0.31 | < 0.001 |
| PC 16:1_20:5 | 0.30 | < 0.001 |

n=120, r ≥ |.30|. PE P, plasmenyl phosphatidylethanolamine; PE, phosphatidylethanolamine; FFA, free fatty acids; PS, phosphatidylserine; PC, phosphatidylcholine; CE, cholesteryl ester; PI, phosphatidylinositol; LPE, lysophosphatidylethanolamine; LPC, lysophosphatidylcholine.

**Supplementary Table 2.** Pearson’s correlations of whole blood fatty acids and biomarkers derived from fatty acids associated with intakes of eicosapentaenoic acid (EPA) plus docosahexaenoic acid (DHA) (g/d)

|  | EPA + DHA Intake | |
| --- | --- | --- |
| Medio lipid | r | P-value |
| % n-3 HUFA | 0.63 | < 0.00001 |
| EPA + DHA wt% | 0.57 | < 0.00001 |
| Total n-3 wt% | 0.57 | < 0.00001 |
| Total n-6 / total n-3 | -0.54 | < 0.00001 |
| 22:6n-3 wt% | 0.53 | < 0.00001 |
| EPA + DHA conc | 0.53 | < 0.00001 |
| 20:5n-3 wt% | 0.51 | < 0.00001 |
| Total n-3 conc | 0.49 | < 0.00001 |
| 20:5n-3 conc | 0.48 | < 0.00001 |
| 22:6n-3 conc | 0.48 | < 0.00001 |
| 22:5n-6 wt% | -0.47 | < 0.00001 |
| 22:5n-6 conc | -0.45 | < 0.00001 |
| 22:4n-6 wt% | -0.43 | < 0.00001 |
| 22:4n-6 conc | -0.43 | < 0.00001 |
| 20:3n-9 wt% | -0.35 | < 0.0001 |
| 20:3n-9 conc | -0.35 | < 0.001 |
| 22:5n-3 wt% | 0.30 | < 0.001 |

n=120, r ≥ |.30|. Fatty acids were correlated quantitatively (conc, µg/100µL) and qualitatively (wt%, weight % of total fatty acids). % n-3 HUFA = 100 × (20:3n-3 + 20:5n-3 + 22:5n-3 + 22:6n-3)/(20:3n-6 + 20:4n-6 + 22:4n-6 + 22:5n-6+20:3n-3 + 20:5n-3 + 22:5n-3 + 22:6n-3)

**Supplementary Table 3**. Stepwise linear regression models for predicting dietary ALA intakes from lipidomic species measured in whole blood

| Variables | B | Std. Error | P | Model R^2^ |
| --- | --- | --- | --- | --- |
| *All participants (n=120)* |  |  |  |  |
| (Constant) | .712 | .292 | .016 | .11 |
| TAG 16:0_18:1_22:5 | .057 | .021 | .007 |  |
| PE 18:2_20:5 | .986 | .384 | .011 |  |
| *Women only (n = 68)* |  |  |  |  |
| (Constant) | 1.193 | .424 | .006 | .25 |
| LPE 22:6 | 1.986 | .568 | <.001 |  |
| PC 16:0_20:4 | -.008 | .002 | .001 |  |
| SM d18:0_16:1(OH) | -.456 | .173 | .011 |  |
| Cer-PE d14:1_21:0 | .361 | .119 | .004 |  |
| PS 20:4_22:6 | -.062 | .024 | .014 |  |
| *Men only (n = 52)* |  |  |  |  |
| (Constant) | -4.208 | 1.430 | .005 | .35 |
| PC 18:1_18:1 | .013 | .006 | .030 |  |
| TAG 18:1_18:1_22:6 | 1.245 | .360 | .001 |  |
| PC O-18:0_22:5 | 1.669 | .576 | .006 |  |
| TAG 18:1_20:4_22:6 | -2.720 | 1.121 | .019 |  |
| *No FO suppl (n = 101)* |  |  |  |  |
| (Constant) | .648 | .325 | .049 | .12 |
| PE 18:2_20:5 | 1.167 | .449 | .011 |  |
| TAG 16:0_18:1_22:5 | .059 | .023 | .013 |  |

ALA, alpha-linolenic acid; TAG, triacylglycerol; PE, phosphatidylethanolamine; LPE, lysophosphatidylethanolamine; PC, phosphatidylcholine; SM, sphingomyelin; Cer-PE, ceramide phosphoethanolamine; PS, phosphatidylserine; PC O, plasmanyl phosphatidylcholine; FO suppl, fish oil supplements.

**Supplementary Table 4**. Stepwise linear regression models for predicting dietary total n-3 PUFA intakes from lipidomic species measured in whole blood

| Variables | B | Std. Error | P | Model R^2^ |
| --- | --- | --- | --- | --- |
| *All participants (n=120)* |  |  |  |  |
| (Constant) | 1.114 | .284 | <.001 | .13 |
| PE P-16:0_20:5 | .155 | .044 | <.001 |  |
| TAG 16:0_18:1_22:6 | .069 | .033 | .041 |  |
| *Women only (n = 68)* |  |  |  |  |
| (Constant) | 1.822 | .440 | <.001 | .48 |
| PE P-18:1_20:5 | .182 | .036 | <.001 |  |
| PE P-16:0_20:4 | -.028 | .008 | .001 |  |
| LPE 22:6 | 2.161 | .736 | .005 |  |
| TAG 18:1_18:1_22:6 | -.334 | .088 | <.001 |  |
| TAG 18:1_20:5_22:6 | 6.169 | 2.098 | .005 |  |
| *Men only (n = 52)* |  |  |  |  |
| (Constant) | -1.472 | 1.135 | .201 | .24 |
| TAG 18:1_18:1_22:6 | .606 | .187 | .002 |  |
| PC P-16:0_18:2 | .278 | .107 | .012 |  |
| *No FO suppl (n = 101)* |  |  |  |  |
| (Constant) | 1.007 | .451 | .028 | .18 |
| PE P-18:0_20:5 | .086 | .032 | .009 |  |
| TAG 16:0_18:1_22:6 | .102 | .037 | .007 |  |
| PC 14:0_20:4 | -.298 | .146 | .045 |  |

PUFA, polyunsaturated fatty acids; PE P, plasmenyl phosphatidylethanolamine; TAG, triacylglycerol; LPE, lysophosphatidylethanolamine; PC P, plasmenyl phosphatidylcholine; PC, phosphatidylcholine; FO suppl, fish oil supplements.

**Supplementary Table 5**. Stepwise linear regression models for predicting dietary EPA intakes from lipidomic species measured in whole blood

| Variables | B | Std. Error | P | Model R^2^ |
| --- | --- | --- | --- | --- |
| *All participants (n=120)* |  |  |  |  |
| (Constant) | .125 | .051 | .455 | .48 |
| PE P-16:0_20:5 | .046 | .006 | <.001 |  |
| PE P-18:0_22:4 | -.004 | .002 | .012 |  |
| TAG 14:0_16:0_18:1 | -.007 | .002 | .001 |  |
| PI 16:0_22:6 | .071 | .029 | .017 |  |
| *Women only (n = 68)* |  |  |  |  |
| (Constant) | -.066 | .123 | .594 | .69 |
| PE 16:0_20:5 | .048 | .009 | <.001 |  |
| Cer-PE d14:1_21:0 | -.118 | .031 | <.001 |  |
| CE 20:5 | .000 | .000 | .014 |  |
| PE 16:0_16:1 | -.007 | .003 | .020 |  |
| PC P-16:0_18:2 | .019 | .007 | .009 |  |
| FFA 22:6 | 34.221 | 15.128 | .027 |  |
| *Men only (n = 52)* |  |  |  |  |
| (Constant) | .089 | .063 | .166 | .76 |
| PE P-16:0_20:5 | .102 | .011 | <.001 |  |
| PE 18:1_20:5 | -.213 | .033 | <.001 |  |
| TAG 16:0_16:0_18:1 | -.009 | .002 | <.001 |  |
| LPE 16:0 | .280 | .056 | <.001 |  |
| TAG 18:1_20:5_22:6 | -.867 | .270 | .002 |  |
| PC 14:0_20:5 | .068 | .030 | .028 |  |
| *No FO suppl (n = 101)* |  |  |  |  |
| (Constant) | -.013 | .055 | .808 | .34 |
| PE P-16:0_22:6 | .022 | .003 | <.001 |  |
| PE P-16:0_20:4 | -.003 | .001 | .008 |  |
| TAG 16:0_16:0_18:1 | -.002 | .001 | .026 |  |

EPA, eicosapentaenoic acid; ; PE P, plasmenyl phosphatidylethanolamine; TAG, triacylglycerol; PI, phosphatidylinositol; PE, phosphatidylethanolamine; Cer-PE, ceramide phosphoethanolamine; CE, cholesteryl ester; PC P, plasmenyl phosphatidylcholine; FFA, free fatty acid; LPE, lysophosphatidylethanolamine; PC, phosphatidylcholine; FO suppl, fish oil supplements.

**Supplementary Table 6**. Stepwise linear regression models for predicting dietary DHA intakes from lipidomic species measured in whole blood

| Variables | B | Std. Error | P | Model R^2^ |
| --- | --- | --- | --- | --- |
| *All participants (n=120)* |  |  |  |  |
| (Constant) | .151 | .099 | .129 | .47 |
| PE P-18:0_22:4 | -.006 | .002 | <.001 |  |
| PE P-16:0_22:6 | .025 | .005 | <.001 |  |
| TAG 14:0_16:0_18:1 | -.007 | .002 | .002 |  |
| CE 22:6 | .000 | .000 | .001 |  |
| SM d18:0_16:1(OH) | -.101 | .045 | .028 |  |
| *Women only (n = 68)* |  |  |  |  |
| (Constant) | -.119 | .132 | .370 | .59 |
| PE P-18:1_22:6 | .027 | .008 | .001 |  |
| PE P-18:0_22:4 | -.006 | .002 | .008 |  |
| FFA 20:5 | 5.223 | 1.722 | .004 |  |
| CE 22:6 | .000 | .000 | .013 |  |
| FFA 22:6 | 36.170 | 17.441 | .042 |  |
| *Men only (n = 52)* |  |  |  |  |
| (Constant) | .136 | .074 | .072 | .71 |
| PE P-16:0_20:5 | .120 | .012 | <.001 |  |
| PE 18:1_20:5 | -.246 | .040 | <.001 |  |
| TAG 16:0_16:0_18:1 | -.009 | .002 | <.001 |  |
| LPE 16:0 | .240 | .069 | .001 |  |
| *No FO suppl (n = 101)* |  |  |  |  |
| (Constant) | .114 | .100 | .255 | .35 |
| PE P-16:0_22:6 | .025 | .005 | <.001 |  |
| PE 18:0_20:4 | -.006 | .002 | .003 |  |
| TAG 14:0_16:0_16:0 | -.040 | .018 | .027 |  |
| PC 16:1_20:5 | .095 | .046 | .039 |  |

DHA, docosahexaenoic acid; PE P, plasmenyl phosphatidylethanolamine; TAG, triacylglycerol; CE, cholesteryl ester; SM, sphingomyelin; FFA, free fatty acid; PE, phosphatidylethanolamine; LPE, lysophosphatidylethanolamine; PC, phosphatidylcholine; FO suppl, fish oil supplements.

**Supplementary Table 7**. Stepwise linear regression models for predicting dietary ALA and total n-3 PUFA intakes using all fatty acids variables including calculations measured in whole blood

| Intake | Variables | B | Std. Error | P | Model R^2^ |
| --- | --- | --- | --- | --- | --- |
| *ALA* |  |  |  |  |  |
| All participants (n=120) | (Constant) | .295 | .457 | .520 | .12 |
|  | 18:1n-9 conc | .063 | .016 | <.001 |  |
|  | 16:1n-7 conc | -.329 | .102 | .002 |  |
| Women only (n = 68) | None |  |  |  | - |
| Men only (n = 52) | (Constant) | -.490 | 1.110 | .661 | .09 |
|  | 18:2n-6 conc | .063 | .028 | .028 |  |
| No FO suppl (n = 101) | (Constant) | 2.869 | .940 | .003 | .13 |
|  | 22:5n-3 conc | .643 | .225 | .005 |  |
|  | 24:1n-9 wt % | -.958 | .334 | .005 |  |
|  | 16:1n-7 wt% | -.555 | .254 | .031 |  |
| *Total n-3 PUFA* |  |  |  |  |  |
| All participants (n=120) | (Constant) | 6.083 | 1.744 | <.001 | .16 |
|  | 22:4n-6 wt% | -1.451 | .412 | <.001 |  |
|  | 16:0 wt% | -.167 | .076 | .031 |  |
| Women only (n = 68) | (Constant) | -.316 | .465 | .499 | .28 |
|  | % n-3 HUFA | .072 | .014 | <.001 |  |
| Men only (n = 52) | (Constant) | 14.544 | 3.987 | <.001 | .20 |
|  | 22:4n-6 wt% | -2.319 | .769 | .004 |  |
|  | 16:0 wt% | -.457 | .172 | .011 |  |
| No FO suppl (n = 101) | (Constant) | 6.037 | 2.000 | .003 | .13 |
|  | 22:4n-6 wt% | -1.476 | .476 | .003 |  |
|  | 22:2n-6 conc | 9.458 | 4.188 | .026 |  |
|  | 16:0 wt% | -.176 | .087 | .044 |  |

ALA, alpha-linolenic acid; PUFA, polyunsaturated fatty acids; conc, µg fatty acid/100µL of whole blood; wt%, relative % of total fatty acids; % n-3 HUFA (20:3n-3 + 20:5n-3 + 22:5n‑3 + 22:6n-3)/(20:3n-6 + 20:4n-6 + 22:4n-6 + 22:5n-6 + 20:3n-3 + 20:5n-3 + 22:5n-3 + 22:6n-3) x 100; FO suppl, fish oil supplements.

**Supplementary Table 8**. Stepwise linear regression models for predicting dietary EPA and DHA intakes using all fatty acids variables including calculations measured in whole blood

| Intake | Variables | B | Std. Error | P | Model R^2^ |
| --- | --- | --- | --- | --- | --- |
| *EPA* |  |  |  |  |  |
| All participants (n=120) | (Constant) | -.523 | .081 | <.001 | .39 |
|  | % n-3 HUFA | .022 | .002 | <.001 |  |
| Women only (n = 68) | (Constant) | -.691 | .111 | <.001 | .49 |
|  | % n-3 HUFA | .027 | .003 | <.001 |  |
| Men only (n = 52) | (Constant) | -.123 | .141 | .385 | .39 |
|  | % n-3 HUFA | .018 | .003 | <.001 |  |
|  | 23:0 wt% | -1.382 | .488 | .007 |  |
| No FO suppl (n = 101) | (Constant) | -.418 | .080 | <.001 | .36 |
|  | % n-3 HUFA | .012 | .002 | <.001 |  |
|  | 18:0 conc | .008 | .002 | .003 |  |
|  | 14:1 wt% | -1.166 | .443 | .010 |  |
| *DHA* |  |  |  |  |  |
| All participants (n=120) | (Constant) | -.526 | .089 | <.001 | .38 |
|  | % n-3 HUFA | .023 | .003 | <.001 |  |
| Women only (n = 68) | (Constant) | -.637 | .119 | <.001 | .45 |
|  | % n-3 HUFA | .027 | .004 | <.001 |  |
| Men only (n = 52) | (Constant) | -.541 | .240 | .029 | .51 |
|  | % n-3 HUFA | .021 | .004 | <.001 |  |
|  | 23:0 wt% | -1.769 | .515 | .001 |  |
|  | 18:0 wt% | .038 | .016 | .020 |  |
| No FO suppl (n = 101) | (Constant) | -.670 | .159 | <.001 | .44 |
|  | % n-3 HUFA | .018 | .003 | <.001 |  |
|  | 18:0 conc | .018 | .004 | <.001 |  |
|  | 12:1 wt% | 8.969 | 3.443 | .011 |  |
|  | 18:0 dma conc | -.082 | .030 | .007 |  |
|  | 14:1 wt% | -1.524 | .647 | .020 |  |

EPA, eicosapentaenoic acid; conc, DHA, docosahexaenoic acid; % n-3 HUFA (20:3n-3 + 20:5n‑3 + 22:5n-3 + 22:6n-3)/(20:3n-6 + 20:4n-6 + 22:4n-6 + 22:5n-6 + 20:3n-3 + 20:5n‑3 + 22:5n-3 + 22:6n-3) x 100; wt%, relative % of total fatty acids; conc, µg fatty acid/100µL of whole blood; FO suppl, fish oil supplements; dma, dimethyl acetal.

**Supplementary Table 9**. Stepwise linear regression models for predicting dietary EPA and DHA intakes qualitative individual fatty acids measured in whole blood

| Intake | Variables | B | Std. Error | P | Model R^2^ |
| --- | --- | --- | --- | --- | --- |
| *EPA* |  |  |  |  |  |
| All participants (n=120) | (Constant) | .119 | .050 | .020 | .37 |
|  | 20:5n-3 wt% | .211 | .031 | <.001 |  |
|  | 20:3n-9 wt% | -1.660 | .375 | <.001 |  |
| Women only (n = 68) | (Constant) | .107 | .071 | .134 | .46 |
|  | 20:5n-3 wt% | .256 | .043 | <.001 |  |
|  | 20:3n-9 wt% | -1.946 | .528 | <.001 |  |
| Men only (n = 52) | (Constant) | .301 | .180 | .101 | .43 |
|  | 22:6n-3 wt% | .049 | .032 | .140 |  |
|  | 23:0 wt% | -1.540 | .488 | .003 |  |
|  | 22:5n-6 wt% | -1.387 | .431 | .002 |  |
|  | 22:5n-3 wt% | .224 | .111 | .049 |  |
| No FO suppl (n = 101) | (Constant) | -.003 | .070 | .968 | .26 |
|  | 22:6n-3 wt% | .059 | .014 | <.001 |  |
|  | 22:5n-6 wt% | -.468 | .184 | .013 |  |
| *DHA* |  |  |  |  |  |
| All participants (n=120) | (Constant) | .187 | .133 | .164 | .38 |
|  | 22:6n-3 wt% | .126 | .023 | <.001 |  |
|  | 22:5n-6 wt% | -1.174 | .296 | <.001 |  |
|  | 23:0 wt% | -.770 | .358 | .033 |  |
| Women only (n = 68) | (Constant) | -1.003 | .204 | <.001 | .45 |
|  | 22:6n-3 wt% | .201 | .028 | <.001 |  |
|  | 18:2n-6 wt% | .026 | .008 | .003 |  |
| Men only (n = 52) | (Constant) | .048 | .284 | .865 | .52 |
|  | 22:5n-6 wt% | -1.996 | .397 | <.001 |  |
|  | 23:0 wt% | -1.898 | .521 | <.001 |  |
|  | 22:5n-3 wt% | .348 | .102 | .001 |  |
|  | 18:0 wt% | .040 | .016 | .015 |  |
| No FO suppl (n = 101) | (Constant) | -.886 | .267 | .001 | .38 |
|  | 22:6n-3 wt% | .047 | .025 | .062 |  |
|  | 22:4n-6 wt% | -.161 | .069 | .022 |  |
|  | 18:0 wt% | .045 | .013 | <.001 |  |
|  | 18:1n-7 wt% | .271 | .096 | .006 |  |
|  | 20:5n-3 wt% | .120 | .052 | .022 |  |

EPA, eicosapentaenoic acid; conc, DHA, docosahexaenoic acid; wt%, relative % of total fatty acids; FO suppl, fish oil supplements.

**Supplementary Table 10**. Stepwise linear regression models for predicting dietary EPA and DHA intakes quantitative individual fatty acids measured in whole blood

| Intake | Variables | B | Std. Error | P | Model R^2^ |
| --- | --- | --- | --- | --- | --- |
| *EPA* |  |  |  |  |  |
| All participants (n=120) | (Constant) | .123 | .046 | .009 | .37 |
|  | 20:5n-3 conc | .100 | .015 | <.001 |  |
|  | 20:3n-9 conc | -.794 | .158 | <.001 |  |
| Women only (n = 68) | (Constant) | .124 | .069 | .079 | .44 |
|  | 20:5n-3 conc | .117 | .021 | <.001 |  |
|  | 20:3n-9 conc | -.953 | .239 | <.001 |  |
| Men only (n = 52) | (Constant) | .320 | .137 | .024 | .40 |
|  | 22:5n-6 conc | -.792 | .175 | <.001 |  |
|  | 22:5n-3 conc | .154 | .046 | .002 |  |
|  | 23:0 conc | -.602 | .209 | .006 |  |
| No FO suppl (n = 101) | (Constant) | -.020 | .058 | .737 | .29 |
|  | 22:6n-3 conc | .030 | .006 | <.001 |  |
|  | 22:5n-6 conc | -.218 | .073 | .003 |  |
| *DHA* |  |  |  |  |  |
| All participants (n=120) | (Constant) | .148 | .105 | .162 | .39 |
|  | 22:6n-3 conc | .063 | .010 | <.001 |  |
|  | 22:5n-6 conc | -.531 | .115 | <.001 |  |
|  | 23:0 conc | -.352 | .153 | .023 |  |
| Women only (n = 68) | (Constant) | .059 | .147 | .690 | .42 |
|  | 22:6n-3 conc | .066 | .014 | <.001 |  |
|  | 22:4n-6 conc | -.149 | .036 | <.001 |  |
| Men only (n = 52) | (Constant) | .099 | .163 | .853 | .54 |
|  | 22:5n-6 conc | -.926 | .179 | .545 |  |
|  | 23:0 conc | -.875 | .218 | <.001 |  |
|  | 18:0 conc | .015 | .005 | <.001 |  |
|  | 22:5n-3 conc | .178 | .047 | .002 |  |
| No FO suppl (n = 101) | (Constant) | -.059 | .124 | .633 | .40 |
|  | 22:6n-3 conc | .049 | .009 | <.001 |  |
|  | 22:5n-6 conc | -.215 | .114 | .062 |  |
|  | 18:0 dma conc | -.098 | .033 | .004 |  |
|  | 18:0 conc | .012 | .004 | .005 |  |
|  | 14:1 conc | -.606 | .282 | .034 |  |

EPA, eicosapentaenoic acid; conc, DHA, docosahexaenoic acid; conc, µg fatty acid/100µL of whole blood; FO suppl, fish oil supplements; dma, dimethyl acetal.

**Supplementary Table 11.** Fatty acid composition of whole blood

| Fatty Acid | All (n = 120) | Women (n = 68) | Men (n = 52) |
| --- | --- | --- | --- |
|  |  | *relative weight %* |  |
| 14:0 | 0.60 ± 0.19 | 0.58 ± 0.16 | 0.62 ± 0.22 |
| 16:0 | 21.03 ± 1.26 | 20.91 ± 1.33 | 21.20 ± 1.19 |
| 18:0 | 11.95 ± 1.24 | 11.86 ± 1.06 | 12.07 ± 1.43 |
| 20:0 | 0.38 ± 0.06 | 0.38 ± 0.04 | 0.38 ± 0.07 |
| 22:0 | 0.98 ± 0.13 | 0.99 ± 0.12 | 0.97 ± 0.15 |
| 24:0 | 1.81 ± 0.24 | 1.78 ± 0.21 | 1.86 ± 0.28 |
| Total SFA | 39.86 ± 1.90 | 39.61 ± 1.76 | 40.22 ± 2.01 |
| 16:1n-7 | 1.02 ± 0.41 | 1.04 ± 0.45 | 1.00 ± 0.37 |
| 18:1n-7 | 1.56 ± 0.16 | 1.57 ± 0.17 | 1.55 ± 0.16 |
| 18:1n-9 | 16.19 ± 1.60 | 15.77 ± 1.29 | 16.75 ± 1.82** |
| 24:1n-9 | 2.30 ± 0.34 | 2.36 ± 0.32 | 2.23 ± 0.34* |
| Total MUFA | 21.93 ± 1.72 | 21.60 ± 1.53 | 22.40 ± 1.90* |
| 18:2n-6 | 19.01 ± 2.31 | 19.47 ± 2.26 | 18.42 ± 2.25* |
| 18:3n-6 | 0.20 ± 0.10 | 0.18 ± 0.08 | 0.22 ± 0.12* |
| 20:3n-6 | 1.47 ± 0.31 | 1.50 ± 0.30 | 1.44 ± 0.32 |
| 20:4n-6 | 9.29 ± 1.13 | 9.34 ± 0.97 | 9.22 ± 1.29 |
| 22:4n-6 | 1.06 ± 0.24 | 1.06 ± 0.21 | 1.07 ± 0.27 |
| 22:5n-6 | 0.19 ± 0.06 | 0.19 ± 0.06 | 0.19 ± 0.06 |
| Total n-6 PUFA | 31.51 ± 2.30 | 32.04 ± 1.91 | 30.84 ± 2.56* |
| 18:3n-3 | 0.41 ± 0.14 | 0.41 ± 0.12 | 0.41 ± 0.16 |
| 20:5n-3 | 0.97 ± 0.46 | 0.98 ± 0.42 | 0.96 ± 0.50 |
| 22:5n-3 | 1.27 ± 0.22 | 1.24 ± 0.21 | 1.33 ± 0.23* |
| 22:6n-3 | 3.44 ± 0.77 | 3.59 ± 0.66 | 3.23 ± 0.85* |
| Total n-3 PUFA | 6.11 ± 1.24 | 6.24 ± 1.13 | 5.95 ± 1.36 |
| 20:3n-9 | 0.10 ± 0.04 | 0.09 ± 0.03 | 0.10 ± 0.04 |
| EPA+DHA | 4.41 ± 1.13 | 4.57 ± 0.99 | 4.19 ± 1.26 |
| Total concentration (µg/100µL) | 213 ± 29 | 212 ± 27 | 213 ± 31 |

SFA, saturated fatty acids; MUFA, monounsaturated fatty acids; PUFA, polyunsaturated fatty acids; EPA, eicosapentaenoic acid; DHA, docosahexaenoic acid. Values are mean ± SD. Significantly different than females by independent t-test, *p < 0.05, ** p < 0.001.

**Supplementary Table 12.** Concentrations of whole blood acyl lipid species with sex differences

| Medio lipid | All (n = 120) | Women (n = 68) | Men (n = 52) |
| --- | --- | --- | --- |
|  | *nmol/mL* | | |
| *Glycerophosphocholines* | | | |
| LPC 16:0 | 47.27 ± 10.36 | 44.18 ± 8.99 | 51.30 ± 10.72** |
| LPC 18:0 | 51.52 ± 11.07 | 48.00 ± 9.98 | 56.13 ± 10.80** |
| LPC O-16:0 | 0.93 ± 0.19 | 0.88 ± 0.17 | 1.00 ± 0.20** |
| PC 14:0_18:2 | 6.22 ± 2.50 | 6.65 ± 2.50 | 5.67 ± 2.41* |
| PC 14:0_22:6 | 1.63 ± 0.80 | 1.84 ± 0.79 | 1.35 ± 0.74** |
| PC 16:0_22:6 | 81.90 ± 17.80 | 85.62 ± 15.40 | 77.03 ± 19.62* |
| PC 16:1_18:2 | 20.55 ± 6.32 | 21.55 ± 5.97 | 19.25 ± 6.58* |
| PC 18:0_18:3 | 33.07 ± 7.33 | 34.70 ± 7.29 | 30.95 ± 6.87* |
| PC 18:0_22:5 | 3.64 ± 1.00 | 3.47 ± 0.93 | 3.87 ± 1.06* |
| PC 18:1_18:1 | 218.61 ± 29.33 | 223.26 ± 28.60 | 212.53 ± 29.43* |
| PC 18:1_22:6 | 2.91 ± 0.85 | 3.14 ± 0.85 | 2.60 ± 0.76** |
| PC 18:2_20:5 | 1.05 ± 0.56 | 1.21 ± 0.54 | 0.83 ± 0.52** |
| PC O-18:0_20:4 | 8.13 ± 1.70 | 8.41 ± 1.67 | 7.77 ± 1.69* |
| PC O-18:0_22:5 | 1.33 ± 0.32 | 1.41 ± 0.30 | 1.21 ± 0.31** |
| PC P-16:0_18:2 | 10.12 ± 2.12 | 10.69 ± 2.18 | 9.36 ± 1.80** |
| *Glycerophosphoethanolamines* | | | |
| LPE 16:0 | 0.75 ± 0.24 | 0.69 ± 0.18 | 0.84 ± 0.29** |
| LPE 18:0 | 2.88 ± 0.79 | 2.69 ± 0.64 | 3.14 ± 0.89* |
| LPE 20:5 | 0.13 ± 0.08 | 0.12 ± 0.07 | 0.15 ± 0.10* |
| PE 14:0_22:6 | 0.07 ± 0.07 | 0.09 ± 0.08 | 0.05 ± 0.05* |
| PE 16:0_18:1 | 69.45 ± 13.28 | 65.99 ± 12.96 | 73.99 ± 12.42** |
| PE 18:0_18:1 | 35.70 ± 6.42 | 33.92 ± 6.21 | 38.03 ± 5.97** |
| PE 18:1_18:1 | 3.35 ± 0.92 | 3.59 ± 0.85 | 3.05 ± 0.91* |
| PE P-16:0_20:4 | 45.46 ± 8.95 | 43.30 ± 8.20 | 48.29 ± 9.17* |
| PE P-18:0_20:4 | 110.47 ± 19.49 | 106.60 ± 19.08 | 115.53 ± 19.03* |
| *Phosphosphingolipids* | | | |
| SM d18:0_16:1(OH) | 0.75 ± 0.33 | 0.81 ± 0.31 | 0.67 ± 0.34* |
| SM d18:1_26:1 | 18.05 ± 4.20 | 17.11 ± 3.52 | 19.28 ± 4.70* |
| Cer-PE d14:2_21:0 | 0.26 ± 0.09 | 0.28 ± 0.09 | 0.22 ± 0.09** |
| Cer-PE d15:1_18:0 | 0.14 ± 0.07 | 0.15 ± 0.06 | 0.12 ± 0.07* |
| *Glycerolipids* | | | |
| TAG 16:0_18:1_22:5 | 10.16 ± 4.38 | 9.31 ± 3.94 | 11.28 ± 4.71* |
| TAG 16:0_18:1_22:6 | 6.54 ± 2.96 | 6.01 ± 2.78 | 7.24 ± 3.07* |
| TAG 18:1_18:1_18:2 | 20.90 ± 7.98 | 19.58 ± 7.25 | 22.62 ± 8.63* |
| TAG 18:1_18:2_22:0 | 0.61 ± 0.68 | 0.48 ± 0.33 | 0.77 ± 0.94* |

Concentration values are reported as mean ± SD. LPC, lysophosphatidylcholine; LPC O, plasmanyl lysophosphatidylcholine; PC, phosphatidylcholine; PC O, plasmanyl phosphatidylcholine; PC P, plasmenyl phosphatidylcholine; LPE, lysophosphatidylethanolamine; PE, phosphatidylethanolamine; PE P, plasmenyl phosphatidylethanolamine; SM, sphingomyelin; Cer-PE, ceramide phosphoethanolamine; TAG, triacylglycerol. Significantly different than females by independent t-test, *p < .05, ** p < .001.

**Supplementary Table 13.** Concentrations of whole blood glycerophosphocholine acyl species

| Medio lipid | All (n = 120) | Female (n = 68) | Male (n = 52) |
| --- | --- | --- | --- |
|  | *nmol/mL* | | |
| LPC 14:0 | 1.20 ± 0.40 | 1.25 ± 0.37 | 1.14 ± 0.44 |
| LPC 20:5 | 1.76 ± 0.73 | 1.69 ± 0.65 | 1.85 ± 0.81 |
| LPC 22:6 | 1.16 ± 0.52 | 1.11 ± 0.42 | 1.22 ± 0.62 |
| PC 14:0_20:4 | 1.51 ± 0.75 | 1.61 ± 0.79 | 1.40 ± 0.69 |
| PC 14:0_20:5 | 0.39 ± 0.47 | 0.36 ± 0.33 | 0.44 ± 0.61 |
| PC 16:0_16:0 | 43.28 ± 6.73 | 42.85 ± 6.82 | 43.85 ± 6.64 |
| PC 16:0_18:1 | 226.18 ± 36.29 | 225.62 ± 38.70 | 226.92 ± 33.23 |
| PC 16:0_18:2 | 282.79 ± 35.33 | 288.13 ± 38.62 | 275.80 ± 29.43 |
| PC 16:0_20:4 | 94.83 ± 22.57 | 96.23 ± 22.84 | 92.99 ± 22.31 |
| PC 16:0_20:4(OH) | 0.80 ± 0.60 | 0.76 ± 0.57 | 0.85 ± 0.63 |
| PC 16:0_20:5 | 60.81 ± 15.68 | 61.70 ± 14.07 | 59.65 ± 17.64 |
| PC 16:0_22:5 | 18.69 ± 3.41 | 18.72 ± 3.10 | 18.66 ± 3.82 |
| PC 16:1_20:5 | 0.28 ± 0.37 | 0.30 ± 0.34 | 0.26 ± 0.41 |
| PC 16:1_22:6 | 2.39 ± 1.17 | 2.34 ± 1.08 | 2.46 ± 1.29 |
| PC 18:0_18:1 | 80.31 ± 12.19 | 78.43 ± 10.64 | 82.78 ± 13.67 |
| PC 18:0_20:4 | 63.00 ± 11.29 | 63.88 ± 10.02 | 61.86 ± 12.77 |
| PC 18:0_20:5 | 12.25 ± 4.43 | 12.59 ± 3.90 | 11.81 ± 5.04 |
| PC 18:0_22:6 | 21.61 ± 6.56 | 22.61 ± 5.85 | 20.31 ± 7.23 |
| PC 18:1_20:5 | 0.16 ± 0.08 | 0.17 ± 0.07 | 0.15 ± 0.08 |
| PC 18:2_22:6 | 1.16 ± 0.38 | 1.15 ± 0.37 | 1.18 ± 0.41 |
| PC P-16:0_20:5 | 5.67 ± 1.25 | 5.70 ± 1.25 | 5.62 ± 1.27 |

Concentration values are reported as mean ± SD. LPC, lysophosphatidylcholine; PC, phosphatidylcholine; PC P, plasmenyl phosphatidylcholine.

**Supplementary Table 14.** Concentrations of whole blood glycerophosphoethanolamine acyl species

| Medio lipid | All (n = 120) | Female (n = 68) | Male (n = 52) |
| --- | --- | --- | --- |
|  |  | *nmol/mL* |  |
| LPE 20:0 | 0.83 ± 0.22 | 0.82 ± 0.23 | 0.86 ± 0.22 |
| LPE 22:6 | 0.44 ± 0.12 | 0.43 ± 0.10 | 0.45 ± 0.14 |
| PE 16:0_16:1 | 10.02 ± 4.59 | 10.34 ± 5.05 | 9.61 ± 3.91 |
| PE 16:0_20:4 | 29.89 ± 5.43 | 29.13 ± 5.71 | 30.89 ± 4.92 |
| PE 16:0_20:5 | 5.18 ± 2.50 | 4.94 ± 2.12 | 5.50 ± 2.92 |
| PE 16:0_22:6 | 15.27 ± 3.83 | 15.81 ± 4.03 | 14.56 ± 3.47 |
| PE 16:1_22:6 | 0.10 ± 0.08 | 0.12 ± 0.09 | 0.09 ± 0.06 |
| PE 17:1_20:4 | 3.59 ± 0.82 | 3.59 ± 0.87 | 3.59 ± 0.77 |
| PE 18:0_20:4 | 46.91 ± 7.88 | 45.90 ± 8.12 | 48.24 ± 7.42 |
| PE 18:0_22:6 | 9.09 ± 1.63 | 9.28 ± 1.76 | 8.83 ± 1.42 |
| PE 18:1_20:4 | 40.41 ± 6.85 | 39.95 ± 7.44 | 41.01 ± 6.00 |
| PE 18:1_20:5 | 2.28 ± 0.73 | 2.36 ± 0.72 | 2.17 ± 0.74 |
| PE 18:1_22:6 | 6.03 ± 1.48 | 6.10 ± 1.46 | 5.93 ± 1.53 |
| PE 18:2_20:5 | 0.47 ± 0.24 | 0.43 ± 0.21 | 0.51 ± 0.27 |
| PE 18:2_22:6 | 1.96 ± 0.70 | 1.91 ± 0.71 | 2.04 ± 0.68 |
| PE 19:0_20:3 | 62.01 ± 15.64 | 63.53 ± 17.32 | 60.03 ± 13.04 |
| PE 19:0_20:4 | 88.78 ± 20.99 | 89.18 ± 21.92 | 88.26 ± 19.91 |
| PE 20:4_20:5 | 0.06 ± 0.07 | 0.05 ± 0.07 | 0.07 ± 0.08 |
| PE 20:4_22:6 | 0.74 ± 0.29 | 0.73 ± 0.29 | 0.75 ± 0.30 |
| PE P-16:0_20:5 | 3.90 ± 2.22 | 3.66 ± 2.11 | 4.21 ± 2.34 |
| PE P-16:0_22:5 | 54.84 ± 9.46 | 53.71 ± 9.83 | 56.32 ± 8.84 |
| PE P-16:0_22:6 | 13.57 ± 3.25 | 13.66 ± 2.99 | 13.45 ± 3.58 |
| PE P-18:0_20:5 | 10.94 ± 4.14 | 10.60 ± 3.84 | 11.38 ± 4.50 |
| PE P-18:0_22:4 | 40.59 ± 8.86 | 39.52 ± 8.55 | 41.98 ± 9.15 |
| PE P-18:0_22:5 | 36.87 ± 5.89 | 36.01 ± 5.76 | 38.00 ± 5.92 |
| PE P-18:0_22:6 | 32.83 ± 7.43 | 32.67 ± 6.38 | 33.04 ± 8.68 |
| PE P-18:1_20:5 | 5.14 ± 2.06 | 5.02 ± 2.01 | 5.30 ± 2.12 |
| PE P-18:1_22:6 | 9.35 ± 2.51 | 9.62 ± 2.30 | 9.00 ± 2.75 |
| PE P-20:0_22:6 | 0.80 ± 0.20 | 0.82 ± 0.17 | 0.78 ± 0.24 |

Concentration values are reported as mean ± SD. LPE, lysophosphatidylethanolamine; PE, phosphatidylethanolamine; PE P, plasmenyl phosphatidylethanolamine.

**Supplementary Table 15.** Concentrations of whole blood acyl species of other polar lipids

| Medio lipid | All (n = 120) | Female (n = 68) | Male (n = 52) |
| --- | --- | --- | --- |
|  |  | *nmol/mL* |  |
| FFA 20:5 | 0.02 ± 0.02 | 0.02 ± 0.01 | 0.02 ± 0.02 |
| FFA 22:6 | 0.00 ± 0.00 | 0.00 ± 0.00 | 0.00 ± 0.00 |
| PI 16:0_20:5 | 0.20 ± 0.24 | 0.17 ± 0.22 | 0.23 ± 0.27 |
| PI 16:0_22:6 | 0.97 ± 0.50 | 1.02 ± 0.52 | 0.91 ± 0.48 |
| PI 18:0_20:4 | 42.74 ± 9.28 | 42.40 ± 8.19 | 43.18 ± 10.59 |
| PI 18:0_20:5 | 0.43 ± 0.29 | 0.44 ± 0.30 | 0.42 ± 0.28 |
| PI 18:0_22:6 | 16.72 ± 7.28 | 17.58 ± 7.09 | 15.60 ± 7.44 |
| PS 16:0_20:5 | 0.05 ± 0.07 | 0.05 ± 0.06 | 0.06 ± 0.08 |
| PS 16:0_22:6 | 1.44 ± 0.60 | 1.48 ± 0.66 | 1.39 ± 0.51 |
| PS 18:0_20:4 | 85.01 ± 12.96 | 85.05 ± 12.50 | 84.95 ± 13.67 |
| PS 18:0_20:5 | 4.88 ± 1.36 | 4.82 ± 1.20 | 4.97 ± 1.55 |
| PS 18:0_22:6 | 39.19 ± 9.36 | 39.27 ± 8.58 | 39.10 ± 10.38 |
| PS 18:1_22:6 | 0.40 ± 0.17 | 0.39 ± 0.16 | 0.41 ± 0.18 |
| PS 20:4_22:6 | 5.36 ± 2.40 | 5.27 ± 2.35 | 5.47 ± 2.50 |
| CER d18:1_24:0 | 1.86 ± 0.41 | 1.86 ± 0.39 | 1.85 ± 0.45 |
| CER d18:1_24:1 | 4.34 ± 0.97 | 4.38 ± 0.96 | 4.30 ± 0.98 |
| SM d18:0_16:1 | 270.86 ± 38.11 | 268.41 ± 31.68 | 274.07 ± 45.30 |
| SM d18:0_24:1 | 174.57 ± 25.77 | 170.95 ± 24.33 | 179.30 ± 27.05 |
| SM d18:1_18:0 | 46.50 ± 9.75 | 47.09 ± 8.90 | 45.73 ± 10.79 |
| SM d18:1_22:0 | 113.39 ± 20.12 | 112.89 ± 18.56 | 114.03 ± 22.16 |
| SM d18:1_23:1 | 48.60 ± 7.09 | 48.40 ± 7.31 | 48.86 ± 6.85 |
| SM d18:1_24:1 | 341.34 ± 46.65 | 337.72 ± 45.70 | 346.08 ± 47.89 |
| Cer-PE d14:1_21:0 | 2.84 ± 0.52 | 2.82 ± 0.49 | 2.85 ± 0.58 |
| Cer-PE d14:2_23:0 | 2.31 ± 0.31 | 2.31 ± 0.29 | 2.31 ± 0.34 |

Concentration values are reported as mean ± SD. FFA, free fatty acids; PI, phosphatidylinositol; PS, phosphatidylserine; CER, ceramide; SM, sphingomyelin; Cer-PE, ceramide phosphoethanolamine.

**Supplementary Table 16.** Concentrations of whole blood nonpolar lipid acyl species

| Medio lipid | All (n = 120) | Female (n = 68) | Male (n = 52) |
| --- | --- | --- | --- |
|  |  | *nmol/mL* |  |
| CE 18:1 | 320 ± 59 | 313 ± 49 | 330 ± 69 |
| CE 18:2 | 1306 ± 189 | 1294 ± 160 | 1323 ± 221 |
| CE 18:3 | 194 ± 77 | 191 ± 61 | 198 ± 95 |
| CE 20:4 | 778 ± 172 | 790 ± 154 | 763 ± 194 |
| CE 20:5 | 438 ± 225 | 443 ± 223 | 432 ± 229 |
| CE 22:6 | 223 ± 117 | 236 ± 119 | 205 ± 112 |
| TAG 12:0_18:1_16:1 | 1.42 ± 2.26 | 1.18 ± 1.79 | 1.73 ± 2.75 |
| TAG 14:0_16:0_16:0 | 0.66 ± 0.78 | 0.56 ± 0.65 | 0.80 ± 0.90 |
| TAG 14:0_16:0_18:1 | 6.39 ± 6.77 | 5.67 ± 6.15 | 7.33 ± 7.46 |
| TAG 16:0_16:0_18:1 | 12.20 ± 8.91 | 11.05 ± 8.60 | 13.69 ± 9.17 |
| TAG 16:0_16:1_18:1 | 23.01 ± 14.44 | 21.83 ± 14.15 | 24.56 ± 14.81 |
| TAG 16:0_17:0_18:1 | 2.32 ± 2.35 | 2.11 ± 2.13 | 2.61 ± 2.61 |
| TAG 16:0_17:1_18:1 | 3.09 ± 2.55 | 2.88 ± 2.34 | 3.35 ± 2.80 |
| TAG 16:0_18:1_18:1 | 48.38 ± 18.80 | 45.97 ± 18.29 | 51.51 ± 19.18 |
| TAG 16:0_18:1_18:2 | 47.64 ± 16.94 | 45.69 ± 16.60 | 50.18 ± 17.20 |
| TAG 16:0_18:2_18:2 | 38.83 ± 17.83 | 36.82 ± 17.42 | 41.46 ± 18.18 |
| TAG 16:0_18:2_20:5 | 2.04 ± 1.48 | 1.85 ± 1.24 | 2.29 ± 1.72 |
| TAG 16:0_22:6_22:6 | 0.28 ± 0.24 | 0.26 ± 0.20 | 0.31 ± 0.28 |
| TAG 16:1_18:2_22:6 | 0.65 ± 0.46 | 0.58 ± 0.37 | 0.73 ± 0.55 |
| TAG 17:0_18:1_18:1 | 3.62 ± 2.32 | 3.41 ± 2.07 | 3.88 ± 2.61 |
| TAG 17:0_18:1_18:2 | 2.69 ± 1.46 | 2.56 ± 1.31 | 2.87 ± 1.64 |
| TAG 18:0_18:1_18:1 | 14.32 ± 7.94 | 13.24 ± 7.15 | 15.72 ± 8.74 |
| TAG 18:0_18:1_18:2 | 22.26 ± 7.92 | 21.21 ± 7.26 | 23.64 ± 8.58 |
| TAG 18:0_18:1_22:6 | 0.25 ± 0.14 | 0.24 ± 0.13 | 0.27 ± 0.16 |
| TAG 18:1_18:1_22:6 | 2.04 ± 1.01 | 2.04 ± 1.01 | 2.03 ± 1.03 |
| TAG 18:1_18:2_22:6 | 2.36 ± 1.36 | 2.25 ± 1.33 | 2.50 ± 1.39 |
| TAG 18:1_20:4_22:6 | 0.38 ± 0.28 | 0.36 ± 0.24 | 0.42 ± 0.33 |
| TAG 18:1_20:5_22:6 | 0.05 ± 0.05 | 0.04 ± 0.04 | 0.05 ± 0.06 |
| TAG 18:1_22:6_22:6 | 0.15 ± 0.16 | 0.16 ± 0.16 | 0.14 ± 0.17 |
| TAG 18:2_18:2_22:6 | 0.68 ± 0.48 | 0.62 ± 0.45 | 0.74 ± 0.51 |

Concentration values are reported as mean ± SD. CE, cholesteryl ester; TAG, triacylglycerol.
